# Multicilin and activated E2f4 induce multiciliated cell differentiation in primary fibroblasts

**DOI:** 10.1038/s41598-018-30791-1

**Published:** 2018-08-17

**Authors:** Seongjae Kim, Lina Ma, Maxim N. Shokhirev, Ian Quigley, Chris Kintner

**Affiliations:** The Salk Institute for Biological Studies, La Jolla, CA 92037 USA

## Abstract

Multiciliated cells (MCCs) are specialized epithelial cells that project hundreds of motile cilia. To form these cilia, MCCs differentiate by dramatically expanding centriole number, using assembly factors required for centriole duplication during the cell cycle and multiple, novel assembly sites, called the deuterosome. The small coiled-coil protein, Multicilin, acting in a complex with the E2F proteins can initiate multiciliated cell differentiation, but reportedly only in a limited range of epithelial progenitors. To examine the nature of this restricted activity, we analyzed Multicilin activity in primary mouse embryonic fibroblasts (MEFs), a cell type distant from the epithelial lineages where MCCs normally arise. We show that Multicilin transcriptional activity is markedly attenuated in MEFs, where it induces only limited centriole expansion in a small fraction of cells. We further show that this transcriptional block is largely bypassed by expressing Multicilin along with a form of E2f4 where a generic activation domain from HSV1 VP16 (E2f4VP16) is fused to the carboxy terminus. MEFs respond to Multicilin and E2f4VP16 by undergoing massive centriole expansion via the deuterosome pathway, recapitulating a temporal sequence of organelle biogenesis that occurs in epithelial progenitors during MCC differentiation. These results suggest that the pattern of organelle biogenesis occurring in differentiating MCCs is largely determined by the transcriptional changes induced by Multicilin.

## Introduction

Centrioles are microtubule-based organelles that serve different functions depending on the phase of the cell cycle^1^. During mitosis, centrioles are core components of the centrosomes that organize the bipolar mitotic spindle required for chromosome segregation. In quiescent cells in G0, the mother centriole converts into a basal body that docks at the plasma membrane and nucleates cilium formation. Since centriole number must be kept constant to ensure proper chromosomal segregation during mitosis, the process of centriole duplication during the cell cycle is tightly regulated. These mechanisms however, can be altered in disease states such as in cancer cells, but also changed during cell differentiation as a means of increasing cilia number.

Centriole duplication occurs during the passage of the cell cycle by the transient activation of a key initiation factor, Plk4, which becomes locally stabilized at a single point at the base of the existing centrioles via scaffolding factors such as Stil, Cep152 and Cep63^2^. Plk4 activation occurs at the G1-S transition, presumably in response to Cyclin/Cdk activity, and the levels of activation are critical for maintaining numerical fidelity^3^. If Plk4 is overexpressed at the G1-S transition, multiple procentrioles can form along the base of the existing centrioles, in a so-called flower arrangement, resulting in supernumerary centriole formation^4–7^. However, the extent of centriole expansion observed in dividing cells is thought to be restricted temporally by the mechanisms governing cell cycle progression but also spatially since it requires existing centrioles as an assembly site. Centriole assembly in the absence of existing centrioles (*de novo*) has also been observed in the cell cycle under certain situations but this pathway is suppressed by the presence of existing centrioles and is relatively slow in nature^7,8^.

In contrast, one of the most dramatic examples of centriole expansion is associated with the differentiation of the vertebrate multiciliated cell (MCC), a specialized epithelial cell that extends hundreds of cilia and produces lumenal flow in several organ systems^9,10^. To extend hundreds of motile cilia, a differentiating MCC requires hundreds of basal bodies, which they assemble after they exit the cell cycle, thus at a stage when centriole formation is normally suppressed in most cell types. Centriole assembly during MCC differentiation employs the same key regulators that underlie centriole duplication during the cell cycle, including Plk4, Cep152, Stil and Sas-6^11–13^. However, only a small fraction (<10%) of this assembly occurs at pre-existing centrioles (where Cep63 is involved)^13^, but instead occurs on a novel structure, called the deuterosome^14^, which forms via Deup1 (a paralog of Cep63)^13^. Multiple deuterosomes arise during MCC differentiation, each capable of nucleating multiple procentrioles, enabling cells to bypass the spatial restrictions that govern centriole duplication during the cell cycle. Centriole assembly during MCC differentiation, however, is not a dysregulated process but one where the various steps of deuterosome formation, new centriole growth, release and maturation are highly ordered^13,15^. These ordered processes require additional levels of regulation that remain poorly defined but may mirror those operating in dividing cells during the centriolar cycle, co-opted to govern centriole expansion during cell differentiation^16^.

MCC differentiation is initiated transcriptionally in the appropriate epithelial progenitors by two related, small coiled-coil proteins, Multicilin (encoded by *Mcidas*) and Gemc1^17–21^. Both proteins are necessary and sufficient to transcriptionally activate MCC differentiation, but differ in how their expression is regulated. Gemc1 appears to be at the top of the hierarchy while Multicilin regulates its own expression in a positive feedback loop, thus reaching the levels of expression required to activate a plethora of genes involved in massive centriole assembly in differentiating MCCs^17–21^. These include genes encoding essentially all known structural components of centrioles, as well as key regulators of centriole assembly, such a*s Plk4*, *Cep152*, *Stil* and *Sass6*^22^. In addition, Multicilin strongly activates *Deup1* transcriptionally (but not the transcription of *Cep63*) to enable centriole assembly on the deuterosome pathway^22^. Finally, Multicilin/Gemc1 also activate the expression of downstream transcription factors, such as *Foxj1*, *Foxn4*, *Tp73*, *Rfx2/3*, and *Myb*, which encompass a gene regulatory network (GRN) that is thought to activate gene expression encoding the hundreds of different proteins involved in multiple, motile cilia formation during MCC differentiation^23–26^.

Multicilin and Gemc1 lack motifs associated with DNA binding but appear to be recruited to DNA by forming a complex with the E2F proteins, either E2f4 or E2f5, along with their heterodimerization partner, Dp1^20,22^. This biochemical interaction along with functional studies of E2f4 leads to the model where Multicilin/Gemc1, recruited to DNA via the E2F proteins, allows MCC gene expression to be turned on^22,27^. However, this model leaves open a number of important issues. First, E2f4/5 have a well-established role in repressing cell cycle gene expression in quiescent cells as part of the DREAM complex^28^. How Multicilin/Gemc1 co-opts these factors to strongly activate a program of gene expression required for MCC differentiation, while other E2F targets involved in cell cycle progression remain off, is still unclear. Second, while Multicilin/Gemc1 can potently activate gene expression required for MCC differentiation, they reportedly only act in a few limited types of progenitors, including those giving rise to the *Xenopus* larval skin, the SVZ in the brain, and the proximal airways^17–19,21^. Because the cell-type, specific factors that enable Multicilin/Gemc1 to drive MCC differentiation in a context dependent manner are unknown, the exact nature of the program that selectively activates gene expression required for this differentiation pathway remains uncertain. Finally, Multicilin and Gemc1 are related to the cell cycle protein, Geminin^29,30^, and effectively drive epithelial progenitors out of the cell cycle^19^. Thus, these proteins conceivably act indirectly by altering the cell cycle in the appropriate epithelial progenitors, thereby enabling an unknown MCC differentiation pathway to unfold. To address these issues, we tested whether Multicilin’s ability to drive MCC differentiation is context dependent, by examining its activity in primary mouse embryonic fibroblasts (MEFs), a heterologous cell type unrelated to the epithelial progenitors that normally give rise to MCC.

Here we show that Multicilin expressed on its own in MEFs is a poor activator of MCC differentiation, and centriole assembly, more specifically. This reduced activity is not due to steady-state instability of Multicilin or to a failure to form a complex with the E2F proteins. However, if Multicilin is expressed along with a form of E2f4 that contains a generic activation domain from HSV1 VP16 (E2f4VP16), the two strongly synergize, effectively activating the expression of key early genes associated with the MCC differentiation program. MEFs respond to Multicilin/E2f4VP16 over a 1–4 day period by largely recapitulating the various steps that occur when mammalian MCC progenitors differentiate in culture, including rapid centriole assembly on existing centrioles, and the formation of deuterosomes, with associated procentrioles in the appropriate number and size. Finally, MEFs expressing Multicilin/E2f4VP16 then switch from centriole assembly to basal body maturation, docking, and extension of multiple motile cilia. We conclude that a transcriptional block normally restricts Multicilin activity in a heterologous cell context, but overcoming this block is sufficient to coordinately drive the appropriate gene expression and set in motion a temporal sequence of organelle biogenesis and maturation that recapitulates the one occurring during MCC differentiation.

## Results

### Multicilin synergizes with activated e2f4 to drive centriole assembly in MEFs

**Figure 1:**
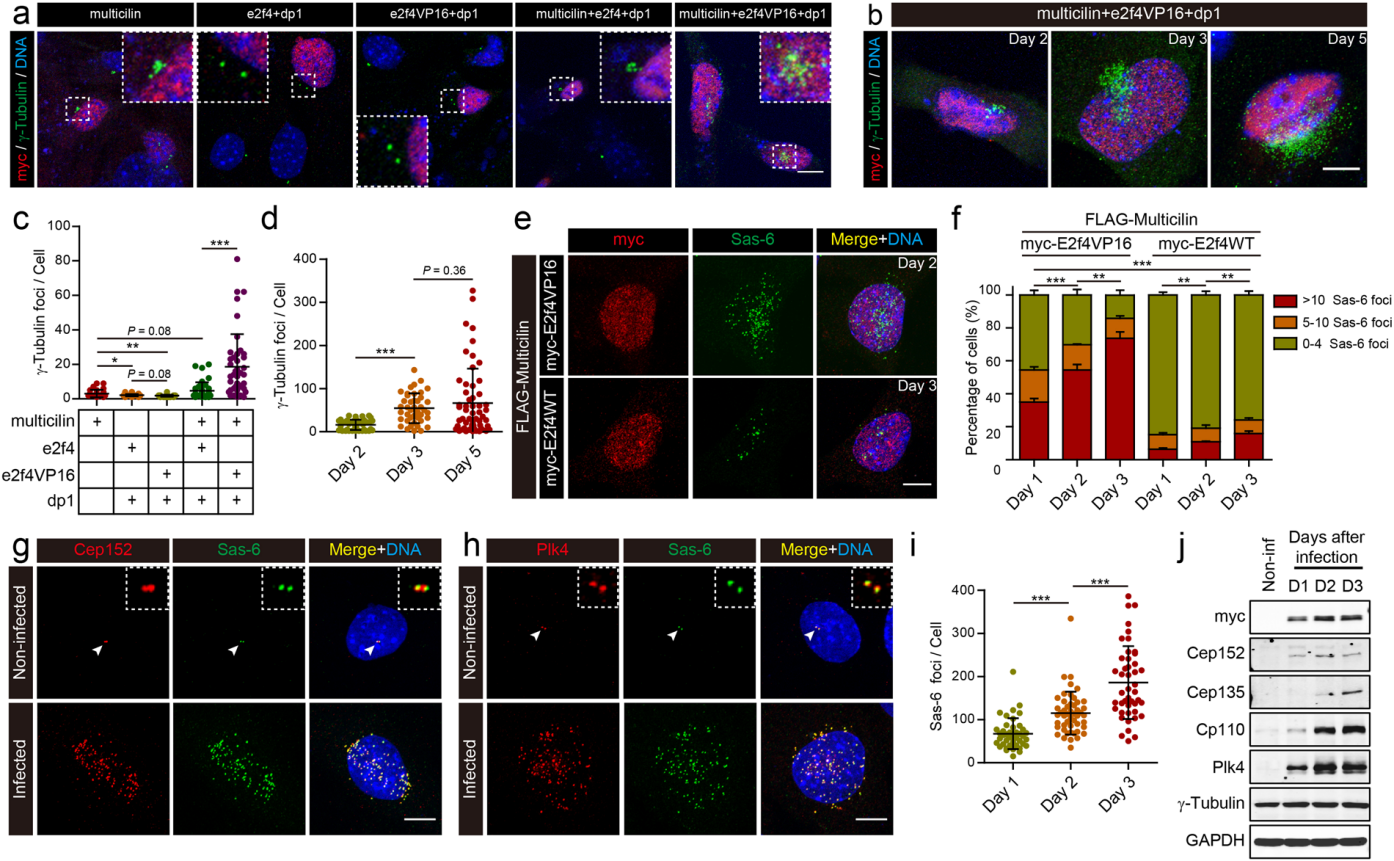
Ectopic expression of multicilin and an activated form of e2f4 in MEFs drives centriole amplification. **(a)** MEFs were transfected as indicated with myc-tagged forms of *Xenopus* multicilin, wild type e2f4, e2f4VP16, and dp1, fixed 2 days post-transfection, immunostained for myc (red), γ-Tubulin (green) and DAPI (blue) and then imaged by confocal microscopy. Insets show magnified images of the centriolar staining. **(b)** MEF cells transfected with myc-tagged forms of multicilin, e2f4VP16, and dp1 were fixed at different days post-transfection, and imaged as in panel a. **(c)** γ-Tubulin foci scored in MEFs transfected with the indicated DNAs and imaged as in panel a. **(d)** Quantification of the experiment shown in panel b. **(e)** MEF cells infected with Ad5 encoding FLAG tagged Multicilin along with myc-tagged E2f4VP16 (2 days PI) or E2f4WT (3 days PI), were fixed and immunostained for the myc-tag (red), and Sas-6 (green), followed by DAPI staining (blue). **(f)** MEFs infected as indicated were scored based on the number of Sas-6 foci, at different days PI. **(g**,**h)** MEFs infected with an Ad5 vector encoding Multicilin/E2f4VP16 or non-infected cells in G2 as controls, were imaged by confocal microscopy 2 days PI, after staining for Cep152 (red) and Sas-6 (green) **(g)** or for Plk4 (red) and Sas-6 (green) (h), followed by DAPI staining (blue). **(i)** Total number of Sas-6 foci in MEFs expressing Multicilin/E2f4VP16 at different days PI. **(j)** Immunoblot analysis of the indicated proteins from non-infected cells or infected cells at different days PI. GAPDH was used as the loading control. Data that are plotted are based on triplicate experiments, where >100 cells were scored at each timepoint. Scale bars = 20 μm **(a)**, and 10 μm **(b,e,g,h)**. Error bars = s.d. Data were compared using a two-tailed *t*-test (*P < 0.05, **P < 0.01, ***P < 0.001, in this and other Figures).

Human MULTICILIN overexpressed in HeLa cells leads to multi-polar spindles and a disruption in mitosis, suggesting defects in centriole biogenesis^30^. To examine this phenotype further, we ectopically expressed *mcidas* in MEFs by transfection, and scored centriole number using γ-Tubulin staining two days later. Multicilin expressed in MEFs caused a small expansion in centriole number in a small fraction of cells, in line with its role in MCC differentiation but in a highly attenuated manner (Figs 1a,c, S1a). To explore the nature of this attenuation, we co-expressed multicilin along with its binding partners, e2f4 and dp1, but found little change (Figs 1a,c, S1a). We next exploited a form of e2f4 where the c-terminal 140 amino acids were replaced with a generic transactivation domain of HSV1 VP16 (amino acids 413–490, hereafter referred to as e2f4VP16)^22^. We found in previous experiments that e2f4VP16 expressed in *Xenopus* skin progenitors did not alter cell proliferation or cell fate but significantly increased basal body number that formed during MCC differentiation (from 160 to over 200 on average)^22^. Thus, this and other observations suggest that e2f4VP16 potentiates the activity of the multicilin/e2f4/dp1 complex, and could thereby relieve the transcriptional block to MCC differentiation in MEFs. Indeed, while e2f4VP16 expressed alone in MEFs had no effect on centriole number when scored by γ-Tubulin staining (Figs 1a,c, S1a), a dramatic synergistic effect occurred when e2f4VP16 and multicilin were expressed together: centriole number expanded massively within 2 days after transfection (Figs 1a–c, S1a) and continued to expand further over the next three days (Figs 1b,d, S1b), following a similar time course reported for centriole amplification in lung progenitors^12,13,31^.

We further documented this synergistic result in MEFs by using an adenovirus vector (pAd/CMV/V5, referred to hereafter as the Ad5 vector) to express a FLAG-tagged form of mouse Multicilin, either alone, or fused, with an intervening T2A cleavage site, to wildtype mouse E2f4, or to mouse E2f4 bearing an VP16 activation domain, identical in design to the activated form of *Xenopus* e2f4 used above (Fig. S2a). Western analysis of MEFs infected with these viruses showed that they expressed Multicilin within two days after infection (Fig. S2c), that the fusion protein is efficiently processed into the two components (Fig. S2c), and that these components interact in a complex, based on immunoprecipitation (Fig. S3a,b). MEFs infected with viruses expressing just Multicilin, alone or with wildtype E2f4, showed a weak expansion in centriole number (based on γ-Tubulin or Sas-6 staining) with only a small fraction of cells (<10%) responded significantly by increasing centriole number even when examined 3 days of infection (Figs 1e,f, S2b,d). In contrast, centriole number in MEFs expressing both mouse Multicilin and E2f4VP16 expanded dramatically over a period of three days in most infected cells (Figs 1e,f,i, S2b,d).

MEFs infected with Multicilin/E2f4VP16 also went on to form numerous foci that stain with markers indicative of new centriole biogenesis^5,32^, including Cep152 (Fig. 1g) and Plk4 (Fig. 1h), key regulatory centriole initiation factors^11,13^ and their downstream target Sas-6, a component of the cartwheel that templates new centrioles (Fig. 1e,h)^33,34^. Additional markers of centriole biogenesis^32^ appeared in the Multicilin/E2f4VP16 expressing MEFs in similar numerous foci, including Centrobin (Fig. S3c,d) and Cep135 (Fig. S3e), markers of the proximal centriole^35,36^; Cp110 (Fig. S3f), a component of the distal centriole^37^; and both Pcnt (Fig. S3g) and Cep215 (Fig. S3h), pericentriolar materials^38,39^. Western blot analysis of both centriole assembly factors and centriolar components also showed a marked increase in levels in infected versus uninfected control MEFs (Fig. 1j). The levels of Plk4 protein induced by Multicilin and E2f4VP16 (Fig. 1j) were particularly impressive since this unstable protein is normally difficult to detect in wildtype cells unless mutated to remove potent degrons^6^. Thus, MEFs respond to Multicilin and E2f4VP16 by markedly increasing centriole assembly factors and structural proteins, resulting in a massive expansion in centriole number. The centriole expansion produced by Multicilin was much more rapid, occurred in many more cells, and reached a much higher level, overall when in the presence E2f4VP16 versus wildtype E2f4. In addition, Multicilin, in the presence of E2f4VP16 but not wildtype E2f4 also strongly activated the expression of target genes, *Foxj1* and *Tp73*, which mediate the ciliogenesis arm of MCC differentiation (Fig. S2c). These findings indicate that the block in MEFs to Multicilin activity can be overcome by potentiating the transcriptional activity of E2f4, suggesting that attenuation in response to Multicilin observed in MEFs is primarily transcriptional in nature.

### Multicilin and E2f4VP16 activate deuterosome-mediated centriole assembly in MEFs

We next asked whether the centriolar expansion initiated in MEFs by Multicilin/E2f4VP16 recapitulates that which occurs during MCC differentiation. Accordingly, infected cells were analyzed at different days PI, using super-resolution microscopy, after staining for proteins that distinguish between centrioles forming on deuterosomes (the so-called DD pathway), marked with Deup1 (Fig. S4b), versus those that form on existing centrioles marked with Cep63 (the so-called MCD pathway) (Fig. S4a)^13^.

**Figure 2:**
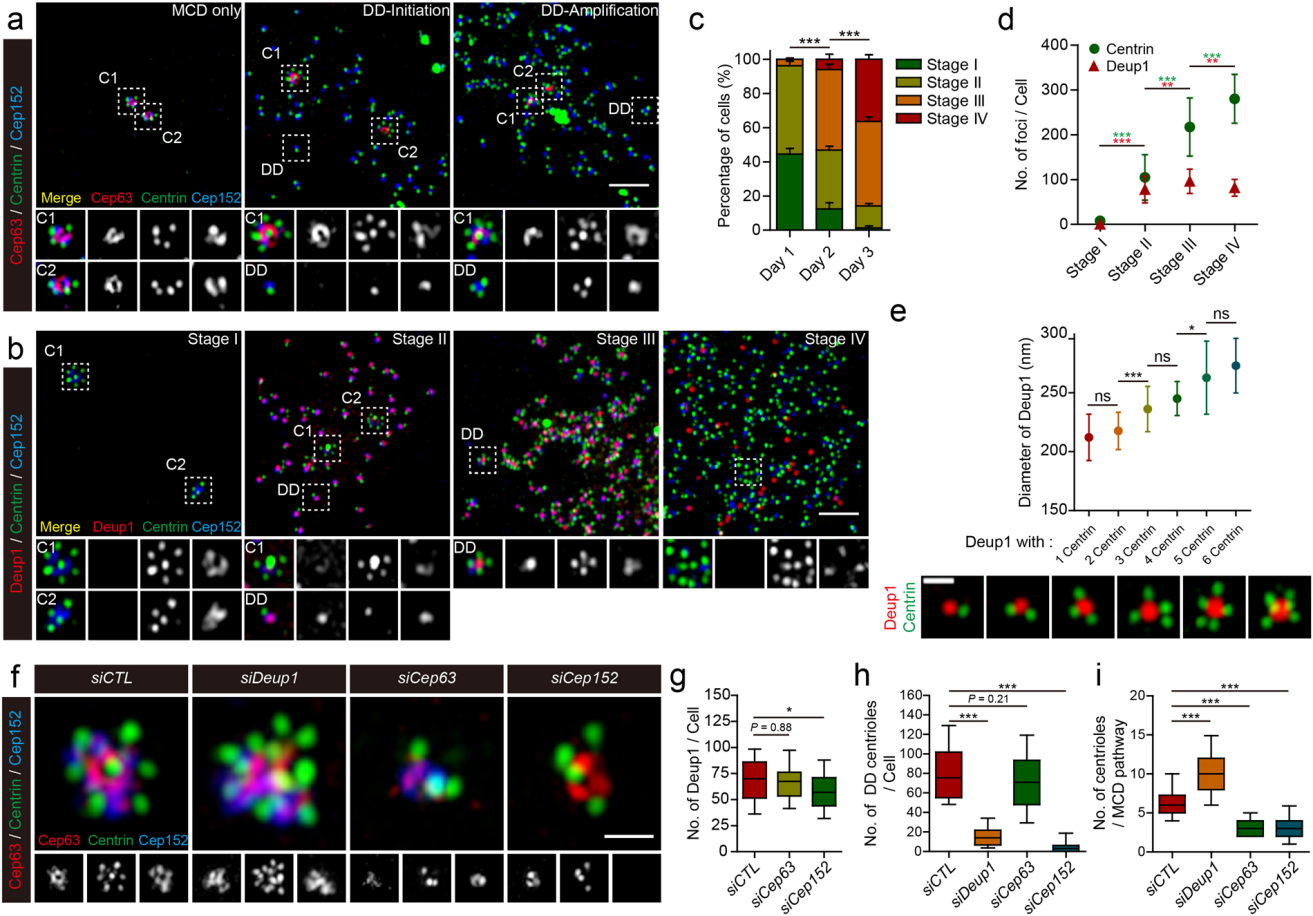
Super-resolution imaging of centriole amplification in MEFs expressing Multicilin and E2f4VP16. **(a)** Super-resolution images of MEFs expressing Multicili/E2f4VP16 stained with Cep63 (red), Centrin (green) and Cep152 (blue). The magnified insets show details for centriole assembly on existing centrioles (C1 or C2, marked with Cep63) versus that occurring at the deuterosome (DD). **(b)** Super-resolution images of MEFs expressing Multicilin/E2f4VP16 stained with Deup1 (red), Centrin (green) and Cep152 (blue). The magnified insets show details for centriole assembly on existing centrioles (C1 or C2, marked with Centrin at the core) versus that occurring at the deuterosome (DD, marked with Deup1). Cells were divided into 4 stages as described in the text. **(c)** MEFs expressing Multicilin/E2f4VP16 were scored at different days PI in triplicate for >100 cells, using the staging of MCC differentiation described in the text. **(d)** Average number of Deup1 and Centrin foci in MEFs expressing Multicilin/E2f4VP16 at different stages of MCC differentiation, based on >35 cells scored at each stage. **(e)** Average size of Deup1 foci based on super-resolution images (Fig. S5a,b and lower panel) of Deup1 (red) and Centrin (green) antibody staining, plotted according to engaged centriole number. **(f)** MEFs were subjected to RNAi knock down (*siCTL*, *siDeup1*, *siCep63* or *siCep152*) 1 day before the infection. MEFs were fixed at day 1 PI (2 days after initial siRNA transfection) then subjected to antibody staining as indicated. Representative super-resolution images of MEFs stained with Cep63 (red), Centrin (green) and Cep152 (blue) followed by cropping to show MCD specific centriole amplification. (**g–i**) Boxplots summarizing the effects of different gene knock downs in MEFs expressing Multicilin/myc-E2f4VP16, on the number of Deup1 foci **(g)**, of Sas-6 foci associated with Deup1 **(h)**, or of procentrioles, marked with Centrin, associated with the pre-existing centrioles marked with Cep63 staining **(i)**. P-values based on a two-tailed, *t*-test. All other error bars = s.d. Scale bars = 2 μm **(a**,**b)** or 0.5 μm **(e**,**f)**.

The MCD pathway was already fully engaged in all MEFs expressing Multicilin/E2f4VP16 at 12 hrs PI, in that the existing mother and daughter centrioles, labeled with Cep63 and Cep152, were often separated from each, and each extended up to 6–7 new centrioles in a flower arrangement, based on Sas-6 staining (Fig. S5a–c, C1, C2). About half of the infected MEFs at 12 hrs PI showed no indication of the deuterosome formation based on Deup1 staining, and we refer to infected MEFs with this pattern of Cep63/Deup1 staining as in stage I (Figs 2b, S5a). Since stage I cells appeared rapidly after infection, and then decreased in frequency as differentiation proceeded (Figs 2c, S5a), we suggest that the MCD pathway is quickly activated in MEFs in response to Multicilin/E2f4VP16 prior to the subsequent appearance of the DD pathway. However, at this and later times, as the DD pathway became more prominent, centrioles at the MCD sites remained attached, and did not increase in number (Fig. 2a,b), suggesting that there was very little, if any, release and re-initiation to amplify centriole number via the MCD pathway even as the DD pathway became active. The rapid formation of new centrioles via the MCD pathway could be due to the rapid activation of centriole assembly factors such as Plk4 that are induced in MEFs by Multicilin/E2f4VP16, as described above, since the pre-existing centriole can act as templates. By contrast Deup1 protein is undetectable in uninfected MEFs, and significant levels need to be induced by Multicilin/E2f4VP16 (Fig. S4c), perhaps delaying the onset of the DD pathway relative to the MCD pathway^13,31,40^.

Deup1 staining consistent with the appearance of the DD pathway was only evident in fraction of the infected MEFs at 12 hours PI, and in all of these cases, the number of Deup1 foci were low in number, and most foci did not appear to contain any procentrioles (perhaps unloaded newly formed deuterosomes), although some extended one, based on Sas-6 staining (Fig. S5b,c). Deup1 staining in these cells was also associated with the pre-existing centrioles (Fig. S5b). At one day PI, or 12 hrs later, about half of the MEFs showed significant engagement of the DD pathway, based on the appearance of numerous Deup1 foci that co-stained for Cep152 and extended a single centriole, as marked by Centrin staining (Fig. 2b–d, referred to as stage II in^40,41^)^13,31^. Each stage II MEF at 1 day PI contained around 80 Deup1 foci on average, and a vast majority of these foci only extended one associated Centrin-labeled procentriole, in a pattern identical to that described in MTECs differentiating into MCCs in primary culture (Fig. 2b,d)^13,31,40,41^. At day 2 PI, the fraction of infected MEFs in stage I dropped dramatically while the fraction in stage II remained constant, suggesting that most MEFs in stage I at day 1 transited into a stage II by day 2 (Fig. 2c). In addition, at two days PI, a major fraction of MEFs could be assigned to a third category (referred to as stage III in^40,41^) where cells displayed a fully engaged MCD pathway, the number of Deup1 foci increased to one hundred on average, and the majority of Deup1 foci contained 2 or more procentrioles as marked with Centrin staining (Fig. 2b–d). This observation suggests that the early phase of the DD pathway in the infected MEFs consists of multiple deuterosomes bearing one procentriole that nucleate additional procentrioles over time^40^. Beginning at day 2 PI and increasing further at day 3 PI, a major fraction of MEFs could be assigned to a fourth category, where the number of Deup1-marked foci begins to drop to 77 on average, the number of Centrin-foci increased further (Fig. 2b–d), and Cep63 staining appears on the centrioles associated with Deup1 foci (data not shown). In MEFs at stage IV, many of the Centrin-foci are no longer associated with the remaining Deup1 foci (Fig. 2b).

Together these observations suggest that centriole assembly via the DD pathway in MEFs expressing Multicilin/E2f4VP16 closely recapitulates how this pathway unfolds in MTECs undergoing MCC differentiation. First, deuterosomes, based on Deup1 staining, rapidly appeared in MEFs expressing Multicilin/E2f4VP16 from essentially few cells with Deup1 foci at 12 hr PI to cells with around 80 deup1 foci on average at 1 day PI (Figs 2c,d, S5c). These deuterosomes have an initial diameter of around 210 nm, grow to reach an average diameter of 270 nm and become more ring-like with time (Figs 2e, S5d–f). A vast majority of the Deup1 foci at early stages of deuterosome formation in infected MEFs contains only one associated centriole on average, but the number of associated Centrin foci increase proportionally at later stages as the size of the deuterosome increases, with an upper limit of 6–7 in total, consistent with the view that larger deuterosomes can nucleate additional centrioles (Figs 2b,e, S5e,f stage III)^13^. However, the average ratio of procentrioles to deuterosome in stage III and IV MEFs is around three, similar to the average ratio observe at peak stages during MCC differentiation in MTECs^13,31^. The total number of Centrin foci peaked at over 200–300 per cell on average, thus within the realm, although at the high end, of basal bodies/cilia number that can form during MCC differentiation in the lung^13^, and more similar to that in the *Xenopus* skin when progenitors express e2f4VP16 (Fig. 2d)^22^. At approximately three to four days PI, the Deup1 foci in cells were arranged in a distinct lattice, and began to decrease in number, as centrioles clearly separated from the Deup1 foci became prominent (Fig. 2b,d).

We further tested the relative contribution of the DD and MCD pathways in MEFs expressing Multicilin/E2f4VP16, by using siRNA knock down to reduce Deup1, Cep63, or Cep152 levels (Fig. S6). Knock down of Deup1 dramatically reduced Deup1 foci formation, and led to a marked reduction in Sas-6 and Cep152 foci that normally formed in large number in MEFs expressing Multicilin/E2f4VP16 (Figs 2h, S6). In contrast, knock down of Deup1 had no apparent effect on centriole assembly via the MCD pathway, but, to the contrary, markedly potentiated the number of centrioles that formed in association with the pre-existing mother and daughter centrioles (Fig. 2f,i). A knock down of Cep63 in MEFs expressing Multicilin/E2f4VP16, while only partial in nature, significantly reduced centriole formation on the pre-existing mother and daughter centrioles, but had no apparent effect on the formation of the Deup1 foci (Figs 2g, S6) or the appearance of new centrioles on these structures (Figs 2h, S6). A knock down of Cep152 in MEFs expressing Multicilin/E2f4VP16 led to a slight reduction in Deup1-marked deuterosomes (Figs 2g, S6) as previously reported in cultured airways progenitors^13^, and a marked reduction in the formation of new Sas-6 and Centrin foci both on the deuterosome (Figs 2h, S6) and on existing mother and daughter centrioles (Figs 2f,i, S6), as expected.

### Centriole formation, basal body maturation and cilia extension are temporally regulated in MEFs in response to Multicilin/E2f4VP16

**Figure 3:**
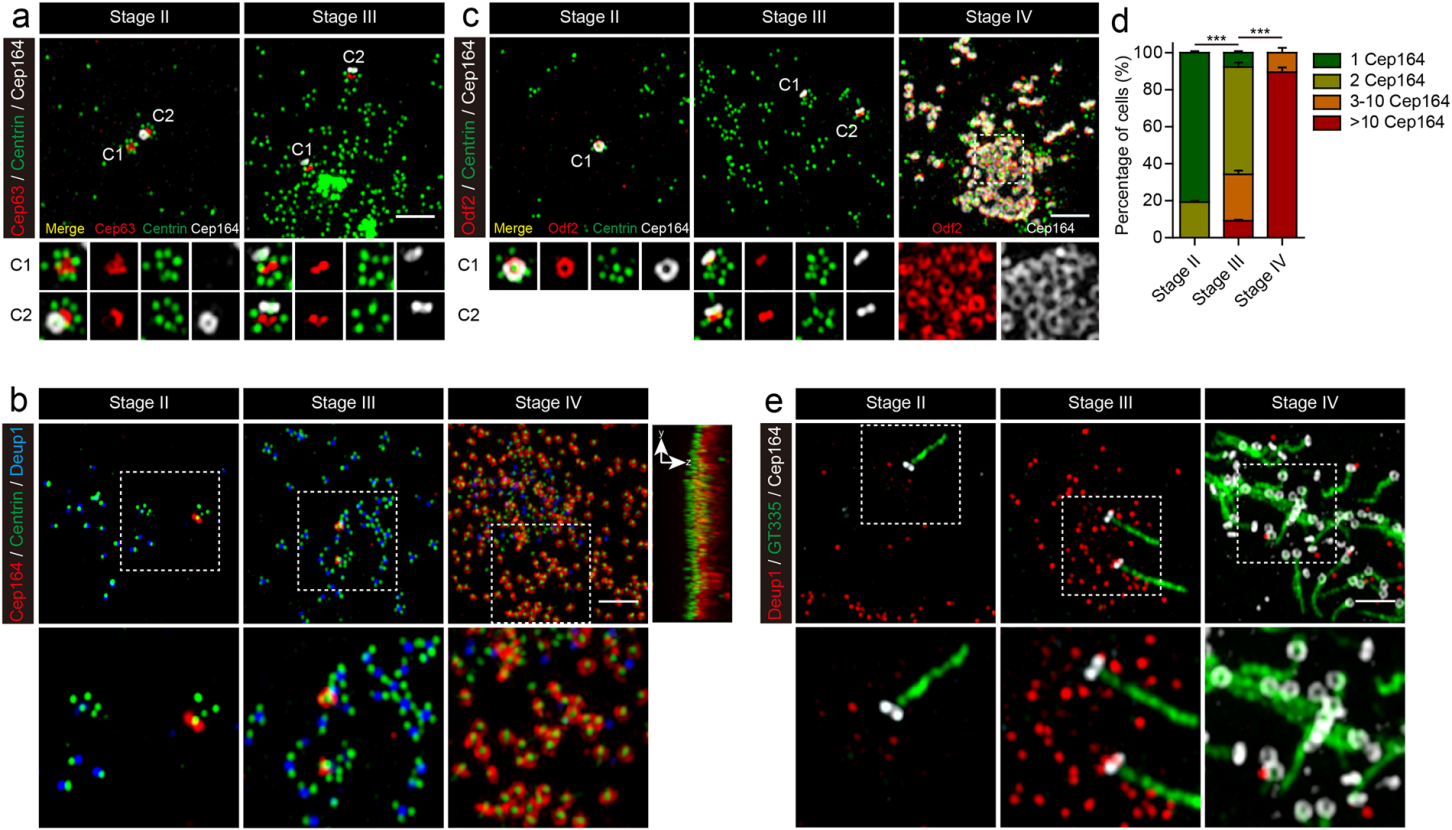
Centriole maturation in MEFs expressing Multicilin and E2f4VP16. **(a)** Super-resolution images of MEFs expressing Multicilin/E2f4VP16 stained for Cep63 (red), Centrin (green) and Cep164 (gray) at different stages of MCC differentiation. Magnified insets show Cep164 staining is first associated with the pre-existing mother centriole (stage II), followed by the daughter centriole (stage III), both marked by Cep63 (C1, C2). **(b)** Super-resolution images of MEFs expressing Multicilin/E2f4VP16, stained for Cep164 (red), Centrin (green) and Deup1 (blue) at different stages of MCC differentiation. The magnified insets show the sequential appearance of Cep164 at the mother centriole, daughter centriole, and finally new centrioles as Deup1 staining disappears. The top and side view of Stage IV with Cep164 (red) and Centrin (green) shows centriole alignment and docking to the surface membrane. **(c)** Super-resolution images of MEFs expressing Multicilin/E2f4VP16, stained for Odf2 (red), Centrin (green) and Cep164 (gray) at different stages of MCC differentiation. The magnified insets show the acquisition of distal appendages (Cep164) and subdistal appendages (Odf2) are closely linked. **(d)** Fraction of infected MEFs containing different extents of Cep164 staining was scored at different stages of MCC differentiation. Only ring-shaped Cep164 staining pattern was counted, scoring >45 cells obtained in triplicate, at each stage. Error bars = s.d. **(e)** Super-resolution images of MEFs expressing Multicilin/E2f4VP16, stained with Deup1 (red), the cilia marker GT335 (green) and Cep164 (gray) at different stages. The magnified insets show that the acquisition of appendages precedes cilia projection. Scale bars = 2 μm.

MEFs expressing Multicilin/E2f4VP16 engage the DD pathway after one day PI, but at three days begin to downregulate this pathway based on the reduction in the number of Deup1 foci and the appearance of Centrin labeled centrioles detached from the deuterosomes (Fig. 2b,d, and Fig. S7; stage IV). This finding indicates that the stepwise nature of centriole biogenesis that occurs during MCC differentiation^13,15^ is largely recapitulated in MEFs in response to Multicilin/E2f4VP16. To address this issue further, we examined a key step during MCC differentiation, when centriole assembly stops, and basal body maturation occurs, by staining for a component of the subdistal appendages, Odf2^42^, or the distal appendages, Cep164^43^ (Fig. 3c,d). Ring-like Cep164 and Odf2 staining was only detected in MEFs at stage I and II at the pre-existing mother centriole that extended a cilium as in uninfected cells (Figs 3a,c; S8a,b; stage II). At stage III, when multiple deuterosomes undergoing centriole assembly were present, the other pre-existing centriole (marked with Cep63) also acquired staining for Cep164 and Odf2 (Figs 3a–c, S8a,b; stage III). At around the stage that the number of Deup1 foci peaked and began to decrease, detached centrioles began prominent, most of these newly synthesized centrioles began to stain with Cep164 and Odf2, indicating that cells undergo a transition between a centriole assembly phase and centriole maturation phase (Fig. 3b,e; stage IV). Super-resolution images first showed spot-like staining with Cep164 and Odf2 on these newly formed centrioles, followed by ring-like structures that then appeared capable of basal body docking and axoneme outgrowth (Figs 3b–e, S8c; stage IV).

**Figure 4:**
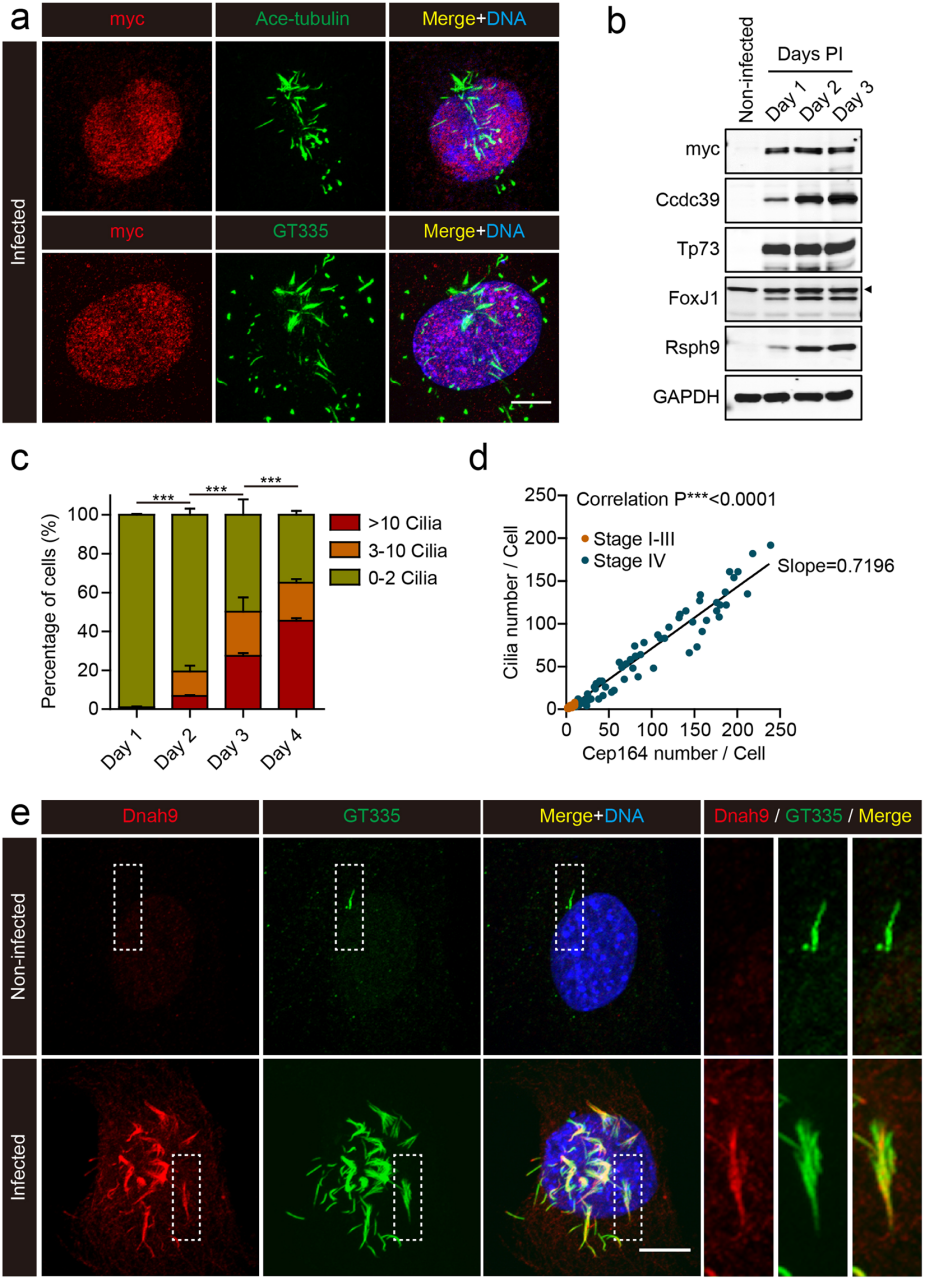
Cilia formation in MEFs expressing Multicilin and E2f4VP16. **(a)** Confocal images of MEFs expressing Multicilin/E2f4VP16, fixed and stained for the myc-tag on E2f4VP16 (red), the cilia markers, acetylated-Tubulin and GT335 (green), followed by DAPI staining (blue). **(b)** Western blot analysis of non-infected MEFs, or MEFs expressing Multicilin/E2f4VP16 with antibodies to myc, Foxj1, Tp73, Ccdc39 or Rsph9, at the indicated day PI. Arrowhead indicates non-specific band. **(c)** The fraction of MEFs expressing Multicilin/E2f4VP16 with the indicated number of cilia based on GT355 staining at the indicated day PI. Error bars = s.d. **(d)** Plot of cilia number per cell versus Cep164 number per cell, taking data from MEFs expressing Multicilin/E2f4VP16 at 1–4 days PI. Cep164 number (matured basal bodies) was scored based on ring-shaped Cep164 detected using super-resolution microscopy and cilia number by Arl13b staining. MEFs classified as stage I-III (as described in the text, orange points) contained few matured centrioles (just the MCD pathway), while those classified as stage IV (dark points) showed matured centrioles based on Cep164 staining that dock and form cilia. Pearson correlation analysis showed a strong correlation between centriole maturation and ciliogenesis with an average slope of 0.7196 indicating that acquisition of appendages precedes ciliogenesis. **(e)** Confocal images of MEFs, either non-infected or expressing Multicilin/E2f4VP16, stained for dynein heavy chain 9 (Dnah9, red), GT335 (cilia marker, green) and DAPI (blue). Magnified insets show MEFs expressing Multicilin/E2f4VP16 extend motile cilia. Scale bars = 10 μm.

As the MEFs expressing Multicilin/E2f4VP16 formed mature centrioles based on Cep164/Odf2 staining, these docked and formed multiple motile cilia (Fig. 4c,d), based on staining with antibodies to acetylated-Tubulin, glutamylated-Tubulin (Fig. 4a), and for DNAH9-Dynein heavy chain (Fig. 4e), the latter a specific marker of motile cilia^44^. Moreover, Western blot analysis shows a marked upregulation in two PCD proteins involved in motile cilia function, Ccdc39^45^, and Rsph9^46^ (Fig. 4b). The initiation of motile cilia formation was likely to be due to marked upregulation of the motile cilia transcription factors (Foxj1 and Tp73, Fig. S9), which occurred in essentially all infected cells. Cilia formation in MEFs was disorganized spatially, perhaps reflecting the fact that MEFs expressing Multicilin/E2f4VP16 retained their mesenchymal morphology (based on a lack of ZO-1 staining), and thus lacked an epithelial apical domain. Nonetheless, these results indicate that Multicilin/E2f4VP16 can induce multiple motile ciliation in a distant embryonic lineage, by inducing a sequence of organelle biogenesis and maturation that closely resembles that which occurs in lung progenitors.

### Multicilin and E2f4VP16 in MEFs induce MCC gene expression

The results above indicate that E2f4VP16 can effectively overcome a transcriptional block in Multicilin activity in MEFs to drive MCC differentiation. Since E2f4 normally acts in MEFs as part of the DREAM complex to repress cell cycle gene expression, this finding raises the question of whether E2f4VP16 overcomes this block by upregulating E2f4 targets in general, or whether it simply allows Multicilin to work more effectively at targets normally expressed during MCC differentiation. To address this issue, we carried out RNAseq analysis on MEFs (Fig. S10a), two days after infection with an Ad5-GFP virus as a control, or with the Ad5 expressing Multicilin and E2f4VP16 (Table S1), to capture the earliest gene responses.

**Figure 5:**
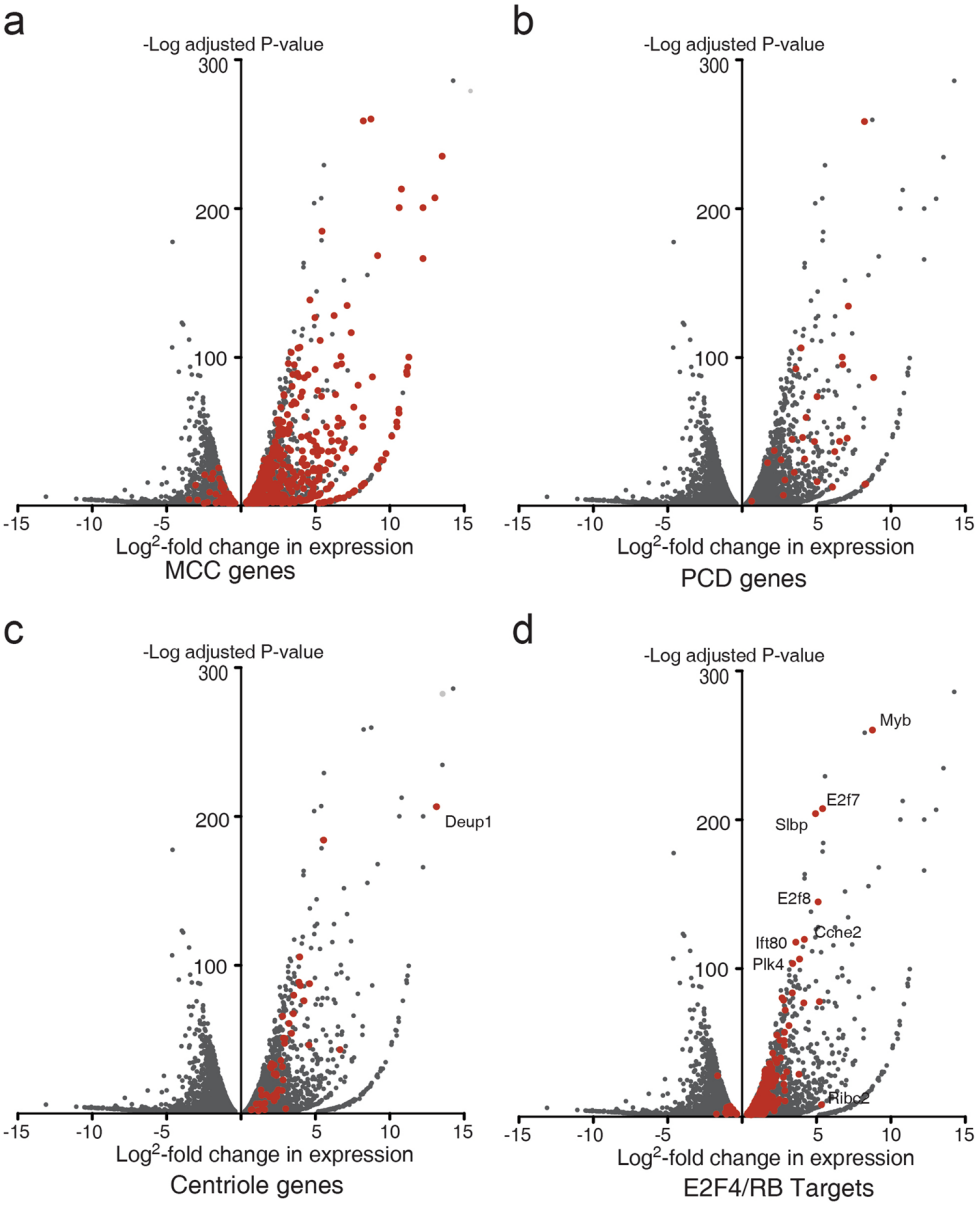
RNAseq analysis of MEFs expressing Multicilin and E2f4VP16. **(a–d)** Shown are volcano plots of genes that are significantly changed (p < 0.05) in expression in MEFs expressing Multicilin/E2f4VP16 versus MEFs expressing GFP, 2 days PI. Both MEFs were serum starved to promote exit from the cell cycle prior to infection. Genes are marked in red that also change during MCC differentiation in *Xenopus* skin progenitors **(a)**, that are mutated in PCD **(b)**^63^, that are associated with centriole biogenesis **(c)**^22^, and that are regulated by E2f4/RB during the cell cycle **(d)**^49^ (Table S1).

The results from the RNAseq analysis indicate that Multicilin/E2f4VP16 causes large changes in gene expression similar to those that occur during normal MCC differentiation. Firstly, MEFs expressing Multicilin/E2f4VP16 versus GFP markedly upregulate gene expression highly enriched for GO terms associated with ciliated cell differentiation, with cilium organization (P < 1E-70), cilium morphogenesis (p < 3E-64), and cilium assembly (p < 1E-63) as the top hits (Table S1). Secondly, of the 814 genes that are associated with MCC differentiation in the Xenopus skin, 340 were upregulated >1.5 fold, P < 0.05 in MEFs expressing Multicilin/E2f4VP16 (Fig. 5a). This core group of genes included those associated with primary ciliary dyskinesia, the human disease caused by motile cilia dysfunction (Fig. 5b, Table S1). Thirdly, a gene regulatory network (GRN) associated with MCC differentiation is strongly upregulated in MEFs expressing Multicilin/E2f4VP16, including *Tp73* (203 fold)^24^, *Foxj1* (112 fold)^25^, *Foxn4* (66-fold)^23^, *Myb* (77-fold)^47,48^, and the RFX family members (*Rfx2*; 27-fold, *Rfx3;* 10-fold)^25^ (Table S1). These transcription factors are the primary regulators of gene expression thought to underlie the formation of motile cilia during MCC differentiation. Finally, MEFs respond to Multicilin-E2f4VP16 by upregulating many of genes required for initiating centriole assembly both in the cell cycle and in multiciliated cells, such as *Plk4* (15-fold), *Cep152* (17-fold), *Sass6* (5.9-fold), *Stil* (8-fold), and *Cep192* (4-fold) (Fig. 5c, Table S1). As during MCC differentiation^22^, Multicilin/E2f4VP16 strongly induces the expression of *Deup1* (170-fold) in MEFs, thus favoring DD-mediated assembly, but has no effect on the transcription of its paralog, *Cep63*, which is used for MCD-mediated assembly (Table S1)^13^. In addition, of the ~500 cell cycle genes identified as potential E2f4/RB targets based on a meta-analysis^49^, a vast majority (390) changed by less than 1.5 fold in MEFs in response to Multicilin/E2f4VP16, while those associated with MCC differentiation are markedly upregulated (Fig. 5d, Table S1). Together these results indicate that Multicilin in a complex with E2f4VP16 retains its selectivity for genes required for MCC differentiation, implying that this core program is sufficient to enable MEFs to undergo MCC differentiation.

The stepwise centriole biogenesis and maturation that occurs in MEFs in response to Multicilin/E2f4VP16, as described above, indicates that Multicilin is also sufficient to transcriptionally induce key regulators required to govern these processes. Consistent with this idea, the RNAseq results show that MEFs respond to Multicilin/E2f4VP16 by strongly upregulating the two genes that flank Multicilin, *Ccno* (68-fold) and *Cdc20b* (183-fold) (Table S1). In addition to *Ccno*, *Ccne1* (18-fold), *Ccne2* (13-fold), and *Cdk1* (8 fold) are also strongly upregulated in MEFs, thus providing the cyclin/CDK regulation that could time the sequence of centriole assembly that occurs during MCC differentiation. Three microRNAs (Mir449a-c) contained within Cdc20b have been shown to regulate the stepwise assembly of centrioles, and these are also strongly upregulated in MEFs by Multicilin/E2f4VP16^50–52^. Finally, both *E2f7* (30-fold) and *E2f8* (26-fold) are among the strongest E2f4/RB targets upregulated in infected MEFs (Fig. 5d), perhaps allowing these cells to constitutively repress cell cycle gene expression that is incompatible with MCC differentiation, particularly that associated with mitosis and cytokinesis^53–55^.

## Discussion

Multicilin and Gemc1 are sufficient to initiate MCC differentiation but only in poised epithelial progenitors, leaving open the question of how direct their action^17–22^. Here we show that Multicilin transcriptional activity is highly attenuated in a non-epithelial cell type, primary MEFs, but that a form of E2f4 bearing a generic activation domain largely overcomes this transcriptional block. Multicilin and E2f4VP16 strongly synergize in MEFs, inducing gene expression required for centriole assembly via the deuterosome pathway, and the downstream transcription factors required for activating gene expression required for motile cilia extension. These results provide strong support for the model that Multicilin recruited to DNA by the E2F proteins is the key step required to activate gene expression during MCC differentiation. In addition, these results indicate that this transcriptional program is sufficient even in MEFs to recapitulate the stepwise sequence of organelle biogenesis that is the hallmark of MCC differentiation.

Expression of Multicilin on its own in MEFs leads to a highly attenuated, but significant expansion of centriole number, mostly like via the MCD pathway. While we have not focused on this response in detail, it highlights the possibility that Multicilin or Gemc1 may normally act to activate gene expression required for centriole biogenesis either during the cell cycle, or in cases where multiple centrioles need to be generated during differentiation, such as in olfactory neurons^56^. In this model, low levels of Multicilin or Gemc1 may be sufficient to activate the transcription of key regulators such as Plk4, Stil, Cep152 and Sas-6, thus enabling centriole duplication or expansion to occur under certain circumstance. This attenuated response, however, is not sufficient for MCC differentiation, likely reflecting the fact that high levels of Deup1 is a key step in driving centriole expansion via the DD pathway^13^.

E2f4 is a component of the DREAM complex with a well-established role in repressing the expression of cell cycle genes in quiescent cells. As part of this complex, E2f4 has been proposed to repress approximately 500 different genes required for cell cycle progression, of which only a small fraction is involved in promoting or regulating the centriolar cycle^49^. Our results show that Multicilin in combination with an activated form of E2f4 can act in MEFs to selectively upregulate gene expression associated with MCC differentiation, while leaving largely untouched the expression of genes involved in other aspects of cell cycle progression. The two proteins partner in a complex that is sufficient to upregulate in MEFs a well-conserved GRN operating during MCC differentiation, including Foxj1, the RFX proteins, Tp73, Foxn4, and Myb^23–25,47,48,57,58^, and gene expression required for centriole expansion via the deuterosome pathway, including Deup1, but not Cep63, key centriole assembly initiation factors (Plk4, Stil, Sas-6, and Cep152), and potential regulatory factors (Cdk1, Ccno, Ccne1 and Ccne2)^11,13,15,16,22,59,60^. Multicilin and activated E2f4 retain their selectivity in MEFs for genes required for MCC differentiation, implying that these factors lie at the core of the transcriptional hierarchy required for MCC differentiation, normally aided in epithelial progenitors by generic activators or kept in check in MEFs by generic repressors. For example, activators such as the grainyhead transcription factors, which operate in the formation of epithelia, could provide a generic epithelial context required for Multicilin transcriptional activity^61^.

A second important finding from this study is that MEFs expressing Multicilin and E2f4VP16 follow the same unidirectional process of DD and MCD centriole assembly that occurs during MCC differentiation. Following infection, new centrioles appear rapidly in MEFs at the base of the existing mother and daughter centrioles, forming a flower-like arrangement that can also arise during the cell cycle when Plk4, or Sas-6 are overexpressed. However, these structures remain intact, and thus restricted in release and re-initiation, limiting the contribution of the MCD pathway to centriole amplification. The deuterosome pathway subsequently becomes prominent in MEFs, following a pattern reported in MTECs^13,40^, wherein numerous Deup1 foci bearing a single procentriole arise simultaneously, growing in size and nucleating additional procentrioles with time. Infected MEFs then transit into a maturation stage, where new centriole formation stops, centrioles are released, and matured based on the appearance of appendage structures (Cep164 and Odf2 staining). At this stage, the Deup1 foci disappear as the newly formed basal bodies dock and extend cilia. The unidirectional nature of this sequence is evident by the fact that when the hundreds of new centrioles are released, additional rounds of new centriole assembly do not occur, even though enhanced levels of Cep152 and Plk4 are still present (Fig. S7). Thus, Multicilin/E2f4VP16 is not only sufficient to initiate organelle biogenesis in MEFs, but also to initiate the regulatory programs that govern it stepwise nature. In addition, the MCD and DD pathways in MEFs follow a similar differentiation sequence, but are not completely in sync, suggesting that this regulatory program can act independently on the state of these two pathways.

Several aspects of MCC differentiation are known to vary in different tissues, reflecting the fact that MCCs are not homogeneous in nature^9^. For example, a lung MCC can extend over a 200 cilia, while MCCs derived from ependymal progenitors extend approximately 50 cilia on average. These differences likely arise by variation in the events that occur during differentiation, including those involved in centriole assembly. Consistent with this idea, lung-derived MCCs (and MEFs expressing Multicilin/Ef4VP16) contain many more deuterosomes (50–100 per cell from stage III-V^13^) compared to the number found in ependymal precursors (~14 deuterosomes/cell)^15^. Deuterosome size and the number of attached procentrioles can also vary between the lung, ependyma and oviduct^9^. In this light, Multicilin/E2f4VP16 appears to be sufficient to produce DD assembly at the upper end of the range (in terms of deuterosome size, number and centriole number), exemplified by MCC differentiation in MTECs. This finding implies that additional regulators beyond Multicilin are unlikely to be required to produce a MTEC-like MCCs, and that in cases were MCC differentiation yields fewer cilia (such as that in ependymal progenitors) the activity of Multicilin may be dampened to drive a variant process (as discussed^41^).

Our results do not address whether E2f4 is also required during MCC differentiation in the cytoplasm to function as part of the deuterosome during centriole assembly, as recently proposed^31^. We note that the synergistic effect that we observe between Multicilin and E2f4VP16 involves forms of E2f4 that are tagged with nuclear targeting motifs. Indeed, we only detect nuclear staining when MEFs expressing Multicilin/E2f4VP16 are stained with an antibody directed against the myc tag on E2f4, or against mouse E2f4 used previously^31^ (data not shown). In addition, the VP16 domain amended to E2f4 that allows MCC differentiation to occur in MEFs in response to Multicilin is known as a generic transcriptional activation domain and unlikely to be fortuitously effective at enhancing the proposed deuterosome function of E2f4. Thus the simplest model to explain our results is one where E2f4VP16 promotes MCC differentiation in MEFs by acting in the nucleus in a transcriptional complex along with Multicilin, although our results do not rule out other potential sites of action.

The high levels of Multicilin and E2f4VP16 achieved in MEFs using the Ad5 vector may be critical in promoting gene expression levels required to assemble hundreds of basal bodies and initiate the formation of multiple motile cilia. These high levels, however, do not appear to accelerate the protracted process of MCC differentiation in mammalian cells implying that rate of organelle biogenesis is set by cell context. As a consequence, by 4–5 days PI in MEFs, cell toxicity becomes an issue, most likely a side-effect of the Adenovirus vector^62^, thus limiting the ability to follow infected cells out to extremely late stages of differentiation. In addition, MEFs are not epithelial cells, and Multicilin and E2f4VP16 expression does not lead to a mesenchymal to epithelial transition (based on ZO-1 staining). The appropriate epithelial cell context is likely required for efficient basal body targeting and docking. If so, MEFs may be useful model for exploring the function of components required for these processes, as a test tube for adding back and testing specific epithelial features.

In sum, these data show that Multicilin along with E2f4VP16 is sufficient to drive the MCC differentiation program in MEFs. This new model of MCC differentiation has a number of advantages in terms of cell availability and accessibility to super-resolution imaging that could be exploited to analysis the mechanisms govern the timing and extent of organelle biogenesis in these cells.

## Methods

### Contact for reagent and resource sharing

Further information and requests for resources and reagents should be directed to and will be fulfilled by the Lead Contact, Chris Kintner (Kintner@salk.edu).

### Cell culture

Freshly prepared primary mouse embryonic fibroblasts (MEFs, strain DR4, non-irradiated) were obtained from Genome Manipulation Core at the Salk Institute. Primary MEFs were grown in DMEM supplemented with 10% FBS and 0.1 mM non-essential amino acid (#11140-050; invitrogen) in a 37 °C humidified incubator with 5% CO_2_. Only MEFs that maintained in culture for less than 4 passages were used in all experiments reported here.

### DNA constructs and transfection experiment

A cDNA clone for myc-tagged *Xenopus laevis* multicilin was described previously (Stubbs *et al*., 2012). A cDNA clone for NLS, 6xmyc-tagged *Xenopus laevis* e2f4 or C-terminus deleted mutant of e2f4 (1–892 bp) fused with VP16 (Viral Protein 16) or *Xenopus laevis* dp1 were described previously (Ma *et al*., 2014). Primary MEFs were plated on 12 mm coverslips with 10% FBS-DMEM for overnight. DNA transfection was performed by using Lipofectamine 2000 according to manufacturer’s instructions. 1 day after DNA transfection, cells were serum-starved with 0.5% FBS-DMEM and fixed according to days after transfection.

### pAd/CMV/V5 expressing mouse multicilin and E2f4 proteins

Gateway entry vectors were used to generate different combinations of mouse Multicilin tagged with 3xFLAG, mouse E2f4 tagged with a NLS and a 6xmyc epitope, and mouse E2f4VP16 tagged with NLS and a 6xmyc epitope, using a combination of PCR along with Topo and Gibson-based cloning strategies. Mouse E2f4 (NCBI Ref: NM_148952.1) was derived from a cDNA obtained from Dharmacon (clone ID 4987691), by using the full-length coding domain (wildtype) or by using a C-terminal truncation (1–774) that was replaced with the transcriptional activation domain of VP16 (Viral Protein 16; amino acids 413–490) from the UL48 gene of Herpes Simplex Virus-1 (HSV-1; GenBank: KM222726.1). Mouse Multicilin (GenBank: AK134107.1) was obtained from the Riken mouse FANTOM clone library (clone ID 5830438C23). The NLS based on SV40 T-antigen (PKKKRKV) and the 6xmyc tags were derived from the CS2 vectors. S2 cleavage sequence used in this study was T2A. Genes assembled in a pENTR/D-TOPO vector (K240020, Invitrogen) were validated by sequencing, and then transferred to the pAd/CMV/V5-DEST vector (#V49320; Invitrogen) using Gateway cloning. The same Ad5 vector expressing GFP was used as a control in the RNAseq analysis.

Adenoviruses stocks were generated by GT3 core at the Salk Institute. Briefly, vector DNAs were initially transfected into 293T cells, verified for intact protein expression by immunoblot and immunostaining, and then used to generate crude adenovirus lysates and titered. Primary MEFs were plated on 12 mm coverslips or proper size of cell dishes with 10% FBS-DMEM for overnight then serum-starved with 0.5% FBS-DMEM for 1 d before adenovirus infection. Adenovirus infection was performed by adding the adenovirus crude lysate to serum-starved MEF cells (50 to 100 MOI) for four hours, washing with pre-warmed PBS once, 0.5% FBS-DMEM once, and then growing infected cells under serum-starved condition with 0.5% FBS-DMEM. The cells were fixed and subjected to analysis according to days post infection (PI).

### Immunoprecipitation and immunoblotting analysis

For detecting protein expression and the interaction between Multicilin and E2f4VP16, MEFs were infected by pAd/CMV/V5 vector encoding NLS-6xmyc-E2f4ΔTA-VP16-T2A-Multicilin. Non-infected MEFs were used as negative control. Two days after infection, the non-infected MEFs or infected MEFs were lysed in buffer (50 mM Tris-Cl [pH 7.5], 420 mM NaCl, 1 mM EDTA [pH 8.0], 5 mM MgCl_2_, 10% Glycerol and 1% Triton X-100) by incubation on ice for 20 min. Lysates were cleared by centrifugation at 12,000 rpm for 20 min at 4 **°**C, and incubated with anti-serum against mouse Multicilin for overnight at 4 **°**C followed by incubation with rProtein G agarose beads (#15920; invitrogen) for 1 h at 4 **°**C. The beads were washed 2 times with lysis buffer and 2 times with wash buffer (50 mM Tris-Cl [pH 7.5], 150 mM NaCl, 1 mM EDTA [pH 8.0] and 1% Triton X-100). Washed beads were treated with LDS sample buffer (#84788; invitrogen) for 5 min at 95 **°**C then supernatant samples were subjected to Western blot with indicated antibodies.

Western blot analysis of Deup1 expression used MEFs infected by the Ad5 vector encoding NLS-6xmyc-E2f4VP16-T2A-Multicilin or non-infected MEFs plated on same day as a control. Two days after infection, the MEF cells were collected in lysis buffer (25 mM Tris-Cl [pH 7.5], 150 mM NaCl, 1 mM EDTA [pH 8.0], 5 mM MgCl_2_ and 1% NP-40), incubated on ice for 20 minutes, cleared by centrifugation at 12,000 rpm for 20 min at 4 °C, incubated with an anti-Deup1 antibody for overnight at 4 °C, and then with rProtein G agarose beads for 1 h at 4 °C. Beads were collected with gentle centrifugation, washed four times with lysis buffer, and bound proteins recovered by heating in LDS sample buffer for 5 min at 95 **°**C. Eluted proteins were subjected to SDS-PAGE, transferred to a membrane that was then blocked with 0.1% casein blocker (#161-0783, Bio-Rad) in 0.2x PBS for 30 min followed by incubation with the indicated primary antibodies for overnight at 4 **°**C in 0.1% Tween-20 in blocking buffer. After extensive washing in 0.1% Tween-20 in PBS, the blots were incubated with Alexa 680 or 800-conjugated anti- mouse or rabbit secondary antibodies (Invitrogen, A21058, A21076, A32735) for 45 min at room temperature, washed with 0.1% Tween-20 in PBS, and imaged using Odyssey (LI-COR).

### Knockdown experiments

MEFs were grown on 12 mm coverslips with 10% FBS-DMEM overnight then serum starved with 0.5% FBS-DMEM. Six hours after serum-starvation, siRNAs were transfected into MEFs using Lipofectamine RNAiMAX (Invitrogen) according to manufacturer’s instructions. The transfection is performed by adding the pre-incubated complex of siRNA with reagent in reduced serum media (Opti-MEM; invitrogen) to cells without media change. Eighteen hours later, the cells were washed with 0.5% FBS-DMEM and infected with Ad5 vector expressing Multicilin/E2f4VP16. Four hours after infection, the cells were washed with pre-warmed PBS once, 0.5% FBS-DMEM once then grown under serum-starved condition with 0.5% FBS-DMEM. Thirty hours after infection, the cells were subjected to analysis by immunostaining. Overall, the cells were knock-downed by siRNAs for >48 hrs and infected for >30 hrs with the Ad5 vector expressing Multicilin/Ef4VP16.

The siRNA for negative control (#12935–113) and siRNAs against Deup1 (MSS214970), Cep63 (MSS282521) and Cep152 (MSS294837) were purchased from Invitrogen. The oligosequences of siRNAs used in this study were siDeup1 (5′-GGG UGA AGU GCA GAC UGC UCA AGA U-3′), siCep63 (5′-AUA UCG AUC UGU UUC AUG AGC UCU U-3′) and siCep152 (5′-CAC CCU CAC UGU ACU UGC CUA UCU U-3′).

### Immunocytochemistry and image processing

MEFS grown on 12 mm coverslips were processed by fixation in methanol at −20 °C for 10 min or in 4% paraformaldehyde in PBS at RT for 10 min, for staining of centriolar proteins or nuclear proteins, respectively. In case of motile cilia staining, cells were pre-extracted with 0.1% Triton X-100 in PBS for 5 sec then, fixed with 4% paraformaldehyde in PEMT (80 mM PIPES (pH6.9), 1 mM EGTA, 1 mM MgCl, 0.5% Triton X-100 in PBS) buffer for 10 min at RT. Cells were washed with PBS, blocked with 3% bovine serum albumin (BSA) in 0.1% PBST for 25 min, and incubated with indicated antibodies for 1.5 h. The cells were washed with 0.1% Triton X-100 in PBS and subsequently incubated with Alexa Fluor 488-, 568-, 647- (Invitrogen) or Cy3-, Cy5-conjugate secondary antibodies (Jackson) using a dilution recommended by the manufacturer. Nuclei were counterstained with DAPI (Sigma). The sample coverslips were mounted in Fluoromount-G (#0100–01; SouthernBiotech) and imaged in a confocal fluorescence microscope (Zei ss, LSM710) with 0.2~0.4 μm thickness of z-stack and processed by using ZEN software (Zeiss, ZEN2011) or ImageJ.

Super-resolution images were taken on the LSM880 Airyscan system (Zeiss) equipped with a Plan Apochromat 63×/1.4 NA oil-immersion objective, 4 laser beams (405, 488, 561 and 633 nm) and 4x zoom from acquisition set. Serial z-stack sectioning was carried out at 120 nm intervals. Airyscan-processing of obtained images was based on a 3D mode with automatic filter strength (~7) using ZEN software (Zeiss), followed by ~20% increased strength from automatically estimated filter strength for better resolution. Airyscan-processed images processed further using ZEN software or ImageJ, ensuring that tandem alterations were made to both experimental and controls.

SR-SIM images were taken on the Elyra PS.1(Zeiss) equipped with Andor iXon3 888 1024 × 1024 9 fps EM-CCD (Electron multiplying charge coupled device) camera, a Plan Apochromat 63×/1.4 NA oil-immersion objective and 3 laser beams (488, 561 and 635 nm). Serial z-stack sectioning was carried out at 125 nm intervals. 3D-SIM image processing of obtained images was done by 3D mode, auto filter strength and baseline cut with manual setting then done with around 20% increased strength from automatically estimated filter strength for better resolution. 3D-SIM processed images were subjected to further processes using ZEN software or ImageJ.

### RNAseq analysis

MEFs (1xE + 6) were plated with 10% FBS-DMEM overnight then starved with 0.5% FBS-DMEM for one day, infected for 4 hours with the Ad5 vector encoding Multicilin/E2f4VP16 (Fig. S3a) or GFP as a control, washed, and then cultured for 48 hrs in 0.5% FBS-DMEM (Fig. S10). Total RNA was extracted and converted into RNAseq libraries following polyA selection, using the Illumina Truseq RNA Sample Preparation kit v2 according to the manufacturer’s instructions, and sequenced on a HiSeq. 2000 or 2500 at 1 × 50 or 1 × 100 base pairs to a depth of 20–40 million reads.

### Bioinformatics

Sequenced reads were quality-tested using FASTQC^64^ and aligned to the mm10 mouse genome using the STAR aligner^65^ version 2.4.0k. Mapping was carried out using default parameters (up to 10 mismatches per read, and up to 9 multi-mapping locations per read). The genome index was constructed using the gene annotation supplied with the mm10 Illumina iGenomes collection^66^ and sjdbOverhang value of 100. Uniquely mapped reads were quantified across all gene exons using the top-expressed isoform as proxy for gene expression with the HOMER^67^ analysis suite, and differential gene expression was carried out with edger^68^ using duplicates to compute within-group dispersion. Differentially expressed genes were defined as having a false discovery rate (FDR) <0.05 and a log2 fold change >1 when comparing two experimental conditions. Table S1 reports normalized (counts per 10 million uniquely mapped reads), log2 fold change, and FDR adjusted p-value. Table S1 reports HOMER functional term overrepresentation of GO biological process, KEGG, Reactome, and Wikipathways with expressed genes corrected for multiple-testing using the Benjamini and Yekutieli general correction for multiple testing^69^.

### Quantification and statistics

The diameter of Deup1 signal was measured as described in Fig. S5. Results are presented as mean ± s.d, unless otherwise indicated. Differences were estimated significant when *P* < 0.05 in an unpaired Student *t*-test using Prism software (two-tailed, Graphpad software). Boxplots represent the 25^th^ and 75^th^ percentiles, the band represents the median, and the ends of the whiskers indicate the 90^th^ and 10^th^ percentiles of the data. Correlation between centriole maturation and ciliogenesis was calculated by a Pearson correlation analysis (two-tailed, Graphpad software). At least three independent experiments were performed for immunostaining or Western blotting analysis followed by quantification or statistical analyses.

## Electronic supplementary material

Supplemental Figures

Table S1

Table S2
