# Supplementary material for: Multicilin and activated E2f4 induce multiciliated cell differentiation in primary fibroblasts
Source: Sci Rep. 2018 Aug 17;8:12369. doi: 10.1038/s41598-018-30791-1 (PMC6098136; doi:10.1038/s41598-018-30791-1)
Supplement: Supplementary file 1 — Supplemental Figures [file 41598_2018_30791_MOESM1_ESM.pdf]

Supplementary Data

Multicilin and activated E2f4 induce multiciliated cell differentiation in primary fibroblasts

Seongjae Kim, Lina Ma, Maxim N. Shokhirev, Ian Quigley, and Chris Kintner

Fourteen Supplemental Figures:

Figure S1: Centriole assembly induced in MEFs by multicilin and the E2f proteins

Figure S2: MEFs expressing mouse Multicilin and the E2f proteins

Figure S3: Centriole assembly in MEFs expressing Multicilin/E2f4VP16

Figure S4: Expression of Cep63 or Deup1 in non-infected MEFs, or in MEFs expressing Multicilin/E2f4VP16

Figure S5: MCD and DD pathway in infected MEFs at 12 hrs PI, and deuterosome size measurements

Figure S6: Super-resolution images of MEFs expressing Multicilin/E2f4VP16 and treated with siRNA to knock-down Deup1, Cep63 or Cep152 expression

Figure S7: Super-resolution images of centriole releasing and clustering at Stage IV in MEFs expressing Multicilin/E2f4VP16

Figure S8: Super-resolution images of centriole maturation in MEFs expressing Multicilin/E2f4VP16

Figure S9: Expression of FoxJ1 and Tp73 in MEFs expressing Multicilin/E2f4VP16

Figure S10: Protocol for RNAseq analysis and cell cycle status

Figure S11-14: Full scans of original blots

Two Supplementary Tables:

Table S1: RNAseq analysis of MEFs expressing Multicilin/E2f4VP16 versus GFP as a control. Sheet 1 shows total normalized reads for all genes, sheet 2 shows GO term enrichment of genes up and downregulated in MEFs expressing Multicilin/E2f4VP16 and sheet 3 annotates genes upregulated in MEFs by Multicilin/E2f4VP16

Table S2: Antibodies used in this study.

Supplemental Figure Legends

Figure S1: Centriole assembly induced in MEFs by multicilin and the E2f proteins

(a) MEFs were transfected with plasmids encoding the indicated proteins, fixed 2 days after transfection and scored for centriole counting using γ-Tubulin staining. Fraction of transfected cells with the indicated γ-Tubulin foci number. **(b)** MEF cells transfected with myc-tagged multicilin and e2f4VP16 were fixed at different days post-transfection, stained for myc (red) to detect transfected cells, for γ-Tubulin (green) to detect centrioles, and DAPI (blue) for DNA. Scale bar=10μm. Data were compared using a two-tailed *t*-test (*P < 0.05, **P < 0.01, ***P < 0.001, in this and other Figures).

Figure S2: MEFs expressing mouse Multicilin and the E2f proteins

(a) Schematics of the Ad5 vectors used to express a 3xFLAG-tagged mouse Multicilin, alone or with an NLS-myc-tagged forms of wildtype E2f4WT (myc-E2f4WT) or E2f4VP16 (myc-E2f4VP16). Proteins were separated by a T2A cleavage sequence. **(b)** MEFs infected with the viruses encoding FLAG-Multicilin alone, or in combination with myc-E2f4WT or with myc-E2f4VP16, were fixed two days PI, and immunostained for γ-Tubulin (red), FLAG-tag (green), and the myc-tag (gray), followed by DAPI staining (blue). Scale bar=10μm. **(c)** Western blot analysis of MEFs 2 days after infection with the indicated viruses, probed with the antibodies against the FLAG or myc-tag, Foxj1, Tp73, and γ-Tubulin as a loading control. **(d)** Dot plot of Sas-6 foci number within MEFs that contain over 10 foci at the indicated day PI. Error bars=s.d.

Figure S3: Centriole assembly in MEFs expressing Multicilin/E2f4VP16

(a) Schematic diagram of the Ad5 vector encoding mouse NLS-myc-E2f4VP16 and Multicilin. A T2A cleavage sequence was used to separate fusion proteins. **(b)** Lysates were prepared from MEFs infected with the virus in panel a, at day 2 PI, and subjected to immunoprecipitation using pre-immune serum (Pre-Im) or a rabbit antibody raised against mouse

Multicilin. A Multicilin antibody recovered both Multicilin and myc-E2f4VP16 using immunoprecipitation, based on Western blot analysis. **(c)** MEFs infected with an Ad5 vector encoding Multicilin and myc-E2f4VP16 or non-infected cells in G2 as controls, were imaged by confocal microscopy 2 days PI, after staining for the myc-tag (red) to identify infected cells, and with mouse antibody against Centrobin (green) to mark newly formed centrioles, and with DAPI (blue) to stain nuclei. **(d)** Total number of Centrobin foci in MEFs expressing Multicilin and myc-E2f4VP16 at different days PI. Error bars=s.d. **(e-h)** Confocal images of MEFs infected with Multicilin and myc-E2f4VP16, or non-infected MEFs in G2 as a control, stained with antibodies against Cep135 (e), Cp110 (f), Pcnt (g), and Cep215 (h), all in red, along with a mouse antibody to Sas-6 (green), followed by DAPI staining (blue). Scale bars=10μm. Insets show a higher magnification of centriolar staining in non-infected cells.

Figure S4: Expression of Cep63 or Deup1 in non-infected MEFs, or in MEFs expressing Multicilin/E2f4VP16

(a, b) MEFs, non-infected or infected with an Ad5 vector encoding Multicilin/E2f4VP16, were fixed at 2 days PI, and stained with antibodies against Cep63 (red, a), Deup1 (red, b), Sas-6 (green, a-b), Cep152 (gray, a-b), and with DAPI (blue). Shown are representative confocal images, using a non-infected MEF in G2 (upper panel). Inserts show a high magnification of centrioles in non-infected MEFs. Scale bars=10μm. **(c)** Western blot analysis of Deup1 expression in non-infected MEFs or MEFs expressing Multicilin/E2f4VP16. At day 2 PI, MEFs were lysed, and the 2 lysates were subjected to immunoprecipitation with the Deup1 antibody followed by Western blotting with the Deup1 antibody. Red arrowhead indicates immunoprecipitated Deup1. Green arrowhead indicates the position of IgG heavy chain.

Figure S5: MCD and DD pathway in infected MEFs at 12 hrs PI, and deuterosome size measurements

(a-c) MEFs infected with an Ad5 vector encoding Multicilin/E2f4VP16, were fixed at 12 hours PI, stained with antibodies against Deup1 (red), Sas-6 (green), and Cep152 (blue), and subjected to super-resolution microscopy. MEFs at this time fell into two stages (a, I and II) based on staining with these markers, carried out in duplicate with a total of >100 cells. Stage I MEFs (upper panels in b and leftside panels in c) showed no detectable Deup1 staining, while the pre-existing centrioles showed a engaged MCD pathway based on multiple attached Sas-6 procentrioles (leftside panels in c). Stage II MEFs (lower panels in b and rightside panels in c) not only showed an engaged MCD pathway, but also low levels of Deup1 staining (lower panels in b), consisting typically of a few foci, some of which were associated with Sas-6 focus (arrows in lower panels in b, D2 in c right side), but others not (arrowheads in lower panels in b, D1 in c right side). Shown are representative super-resolution images. Scale bars=2μm. **(d)** Method for measuring the outer diameter of Deup1 foci, using an approach described previously (1, 2). Super-resolution images of Deup1 foci were captured with an Airyscan microscope. Scale bar=0.2μm. Apparent diameter of each Deup1 focus was calculated by averaging orthogonal distances along points defined by 50% relative intensity. **(e)** Shown are the average mean diameters (microns ± s.d.) of Deup1 foci, according to number of associated centrioles (Centrin foci), along with sample size (n). **(f)** Ring-like deuterosomes form in MEFs expressing Multicilin/E2f4VP16, shown in a super-resolution SIM image, centering on several larger Deup1 foci (red) bearing multiple associated centrioles (Centrin, green). Scale bar=0.5μm.

Figure S6: Super-resolution images of MEFs expressing Multicilin/E2f4VP16 and treated with siRNA to knock-down Deup1, Cep63 or Cep152 expression

(a, b) MEFs were transfected with siRNAs (*siCTL*, *siDeup1*, *siCep63* and *siCep152*) 1 day before infection with the Ad5 expressing Multicilin/E2f4VP16. At day 1 PI (2 days after siRNA

transfection), MEFs were fixed and subjected to immunostaining with Deup1 (red, a) or Cep63 (red, b), Sas-6 (green, a-b) and Cep152 (blue, a-b). Insets (b) show MCD centriole amplification (C1 or C2) in detail. Scale bars=2μm.

Figure S7: Super-resolution images of centriole releasing and clustering at Stage IV in MEFs expressing Multicilin/E2f4VP16

(a) Super-resolution images of stage IV MEFs expressing Multicilin/E2f4VP16 stained with antibodies against Plk4 (red), Centrin (green) and Cep152 (blue). Insets were magnified with grayscale and shown in close up. **(b)** Super-resolution images of stage IV MEFs expressing Multicilin/E2f4VP16 stained with antibodies against Deup1 (red) and Centrin (green). Dotted-lines indicated regions where centriole clustering occurs following release and disappearance of Deup1 foci. Scale bars=2μm.

Figure S8: Super-resolution images of centriole maturation in MEFs expressing Multicilin/E2f4VP16

(a) Super-resolution images of MEFs expressing Multicilin/E2f4VP16 stained with antibodies against Cep63 (red), GT335 (green) and Cep164 (gray), showing an example of stage II, when just the original mother centriole extends a cilium, and stage III, when the original daughter centriole has matured and also extends a cilium. Insets (C1, C2) show the existing mother (C1) and daughter (C2) at higher power. **(b)** Super-resolution images of MEFs expressing Multicilin/E2f4VP16 stained with antibodies against Odf2 (red), GT335 (green) and Cep164 (gray), showing examples of stage II and III as in panel a, as well as stage IV, when most of the centrioles generated by the DD pathway also mature. Insets show the acquisition of distal appendage (Cep164, gray) and sub-distal appendage (Odf2, red) precedes cilia formation. **(c)** Super-resolution images of MEFs at stage IV expressing Multicilin/E2f4VP16 stained with antibodies against Odf2 (red), Centrin (green) and Cep164 (gray). Insets show examples where the appendages proteins

(Cep164 are Odf2) are initially recruited with a dot-like pattern (I, Initial) then apparently matured (M, Matured) into ring-like pattern. Scale bars=2µm.

Figure S9: Expression of FoxJ1 and Tp73 in MEFs expressing Multicilin/E2f4VP16

(a, b) Non-infected MEFs or MEFs expressing Multicilin/E2f4VP16 were fixed 2 days PI, and confocal imaged after staining for the myc-tag (red) and Foxj1 (a) or Tp73 (b) (green), followed by DAPI staining (blue). Scale bars=10µm. **(c)** Quantification of staining shown in panels a and b.

Figure S10: Protocol for RNAseq analysis and cell cycle status

(a) Schematic diagram for RNAseq analysis using MEFs expressing GFP (pAd5-GFP) or *Multicilin/E2f4VP16* (pAd5-Multicilin/E2f4VP16). **(b)** Quantification for validation of cell cycle exit using primary cilia staining at Day 2 PI in control group (pAd5-GFP infected MEFs). Random area is selected then scored the Arl13b positive cells per GFP positive cells as infected cells with cell cycle exit status. **(c)** Representative confocal image of control group (pAd5-GFP infected MEFs) stained with Arl13b (red), GFP (green) and DAPI for DNA (blue). Scale bar=10µm.

Figure S11: Full scan of Western Blot Figure 1j

Figure S12: Full scan of Western Blot Figure 4b

Figure S13: Full scan for Western Blot Figure S2c

Figure S14: Full scan for Western Blot Figures S3b and S4c

References:

1. Al Jord A, et al. (2014) Centriole amplification by mother and daughter centrioles differs in multiciliated cells. Nature 516(7529):104-107.
2. Zhao H, et al. (2013) The Cep63 paralogue Deup1 enables massive de novo centriole biogenesis for vertebrate multiciliogenesis. Nat Cell Biol 15(12):1434-1444.

Kim et al Figure S1

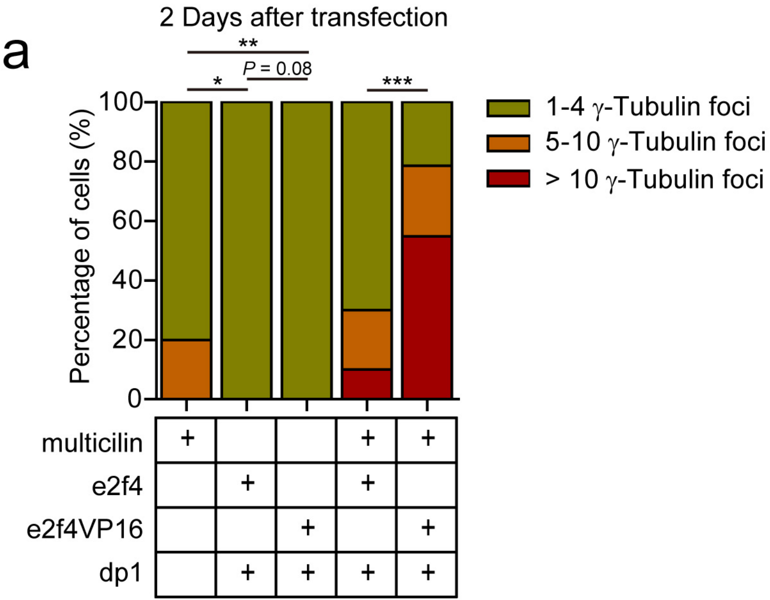

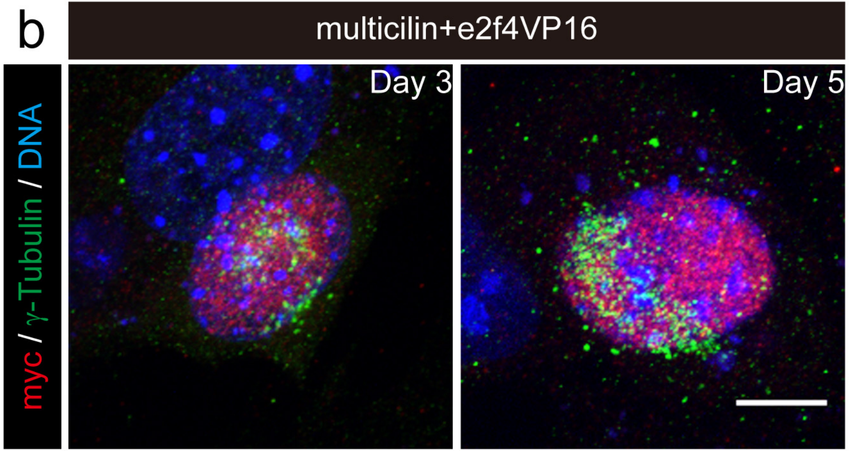

Kim et al Figure S2

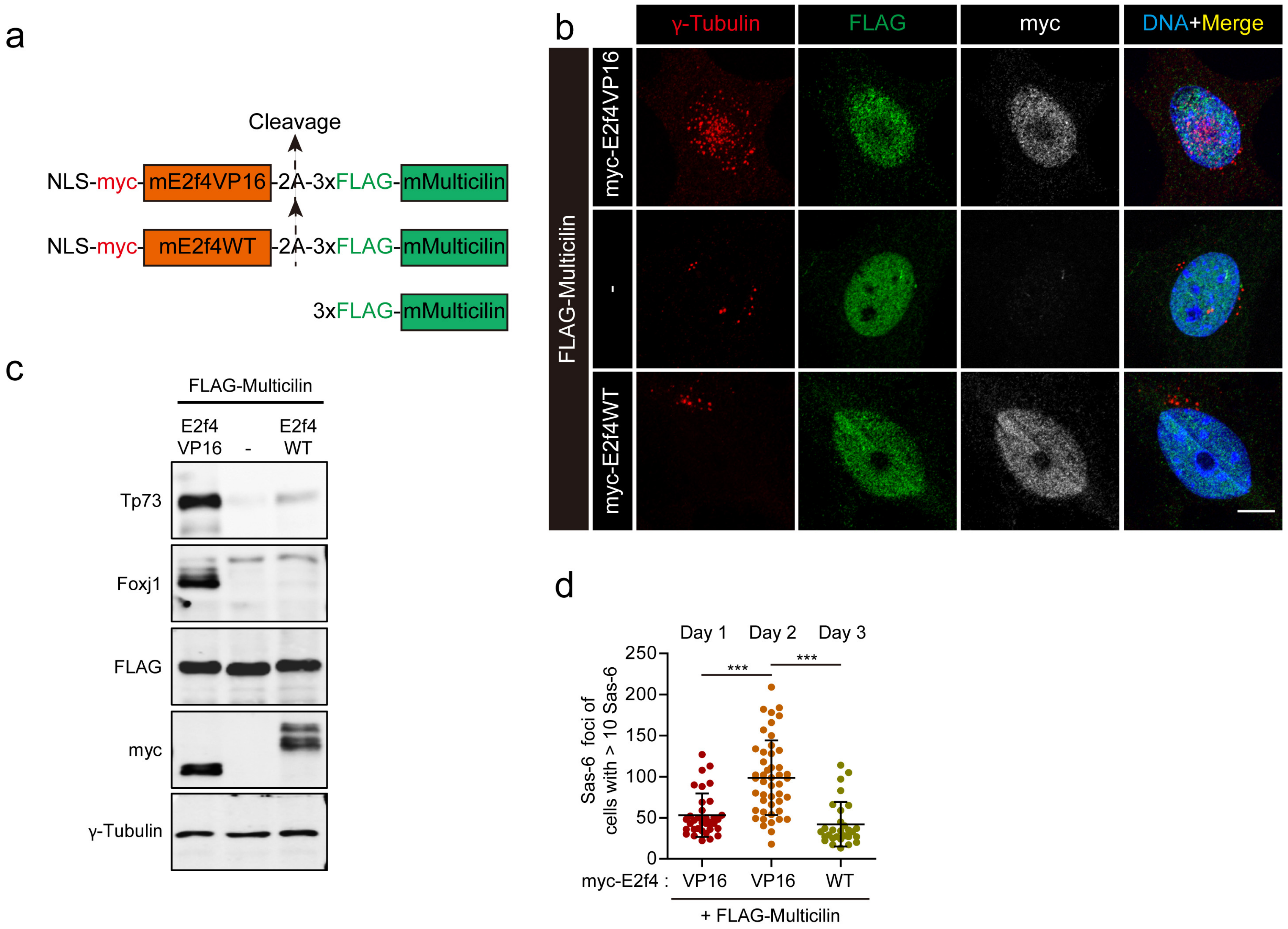

Kim et al Figure S3

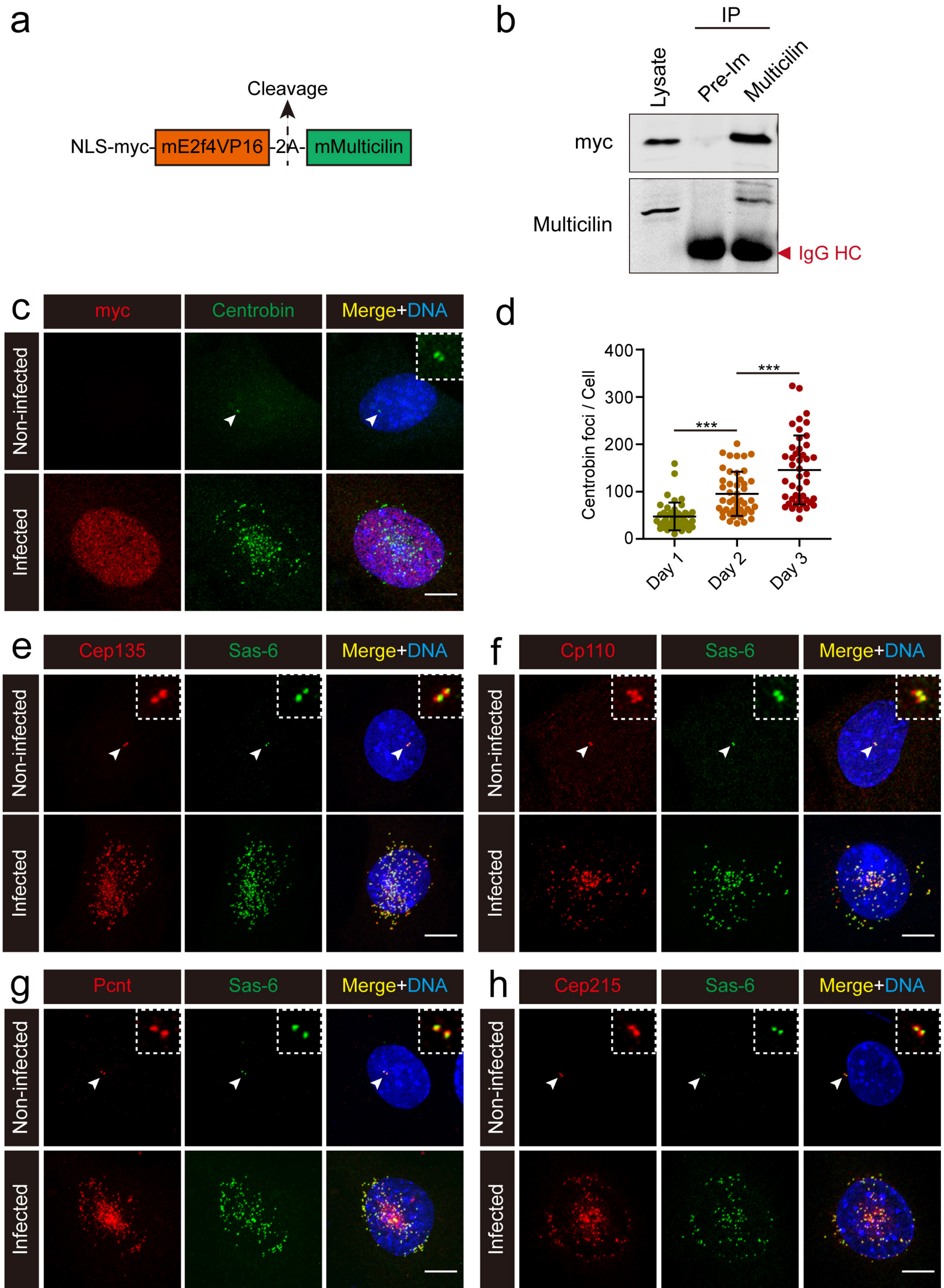

Kim et al Figure S4

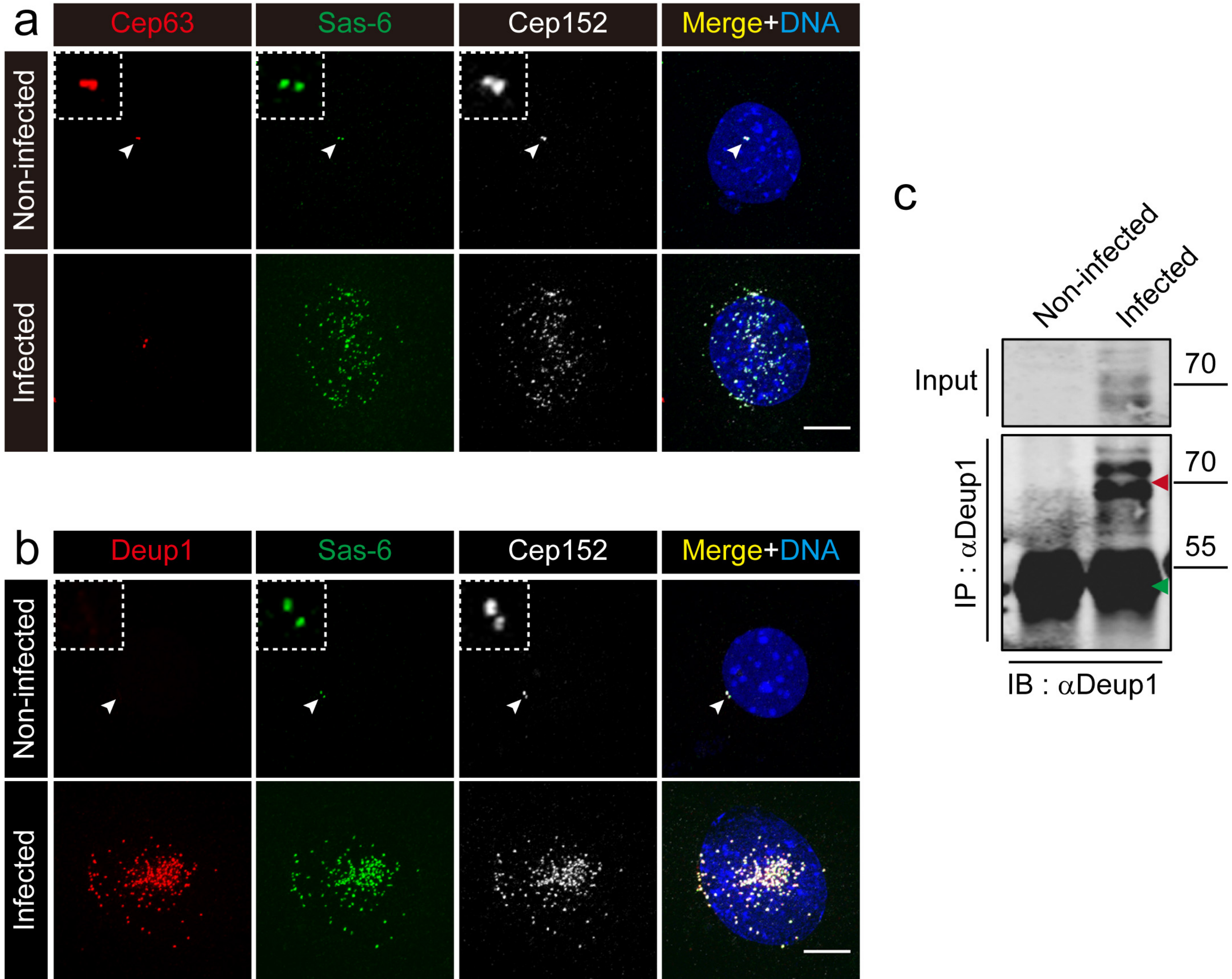

Kim et al Figure S5

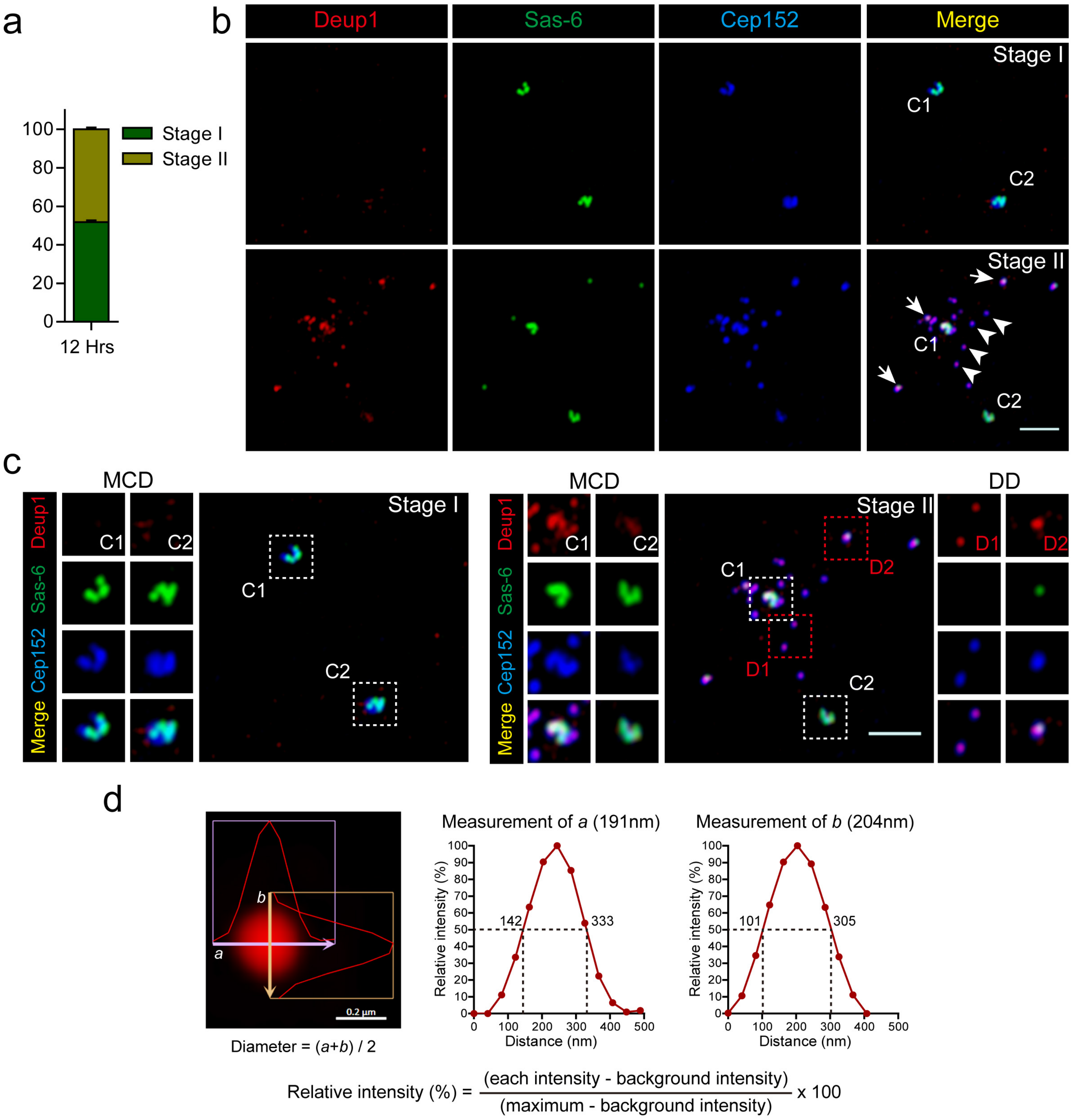

e

	n	No. of Centrin	Diameter of Deup1
Deup1 with	24	1 Centrin	212.0 ± 19.65
	24	2 Centrin	217.6 ± 15.79
	24	3 Centrin	236.2 ± 19.32
	26	4 Centrin	245.1 ± 14.37
	26	5 Centrin	262.8 ± 31.16
	24	6 Centrin	273.2 ± 23.25

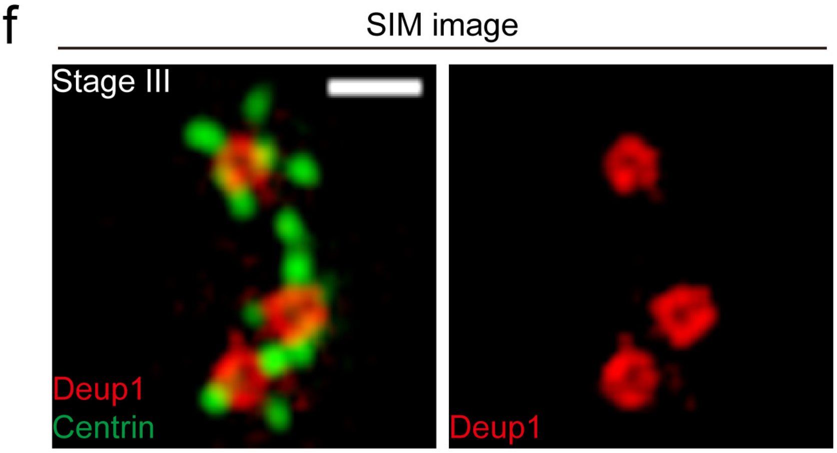

Kim et al Figure S6

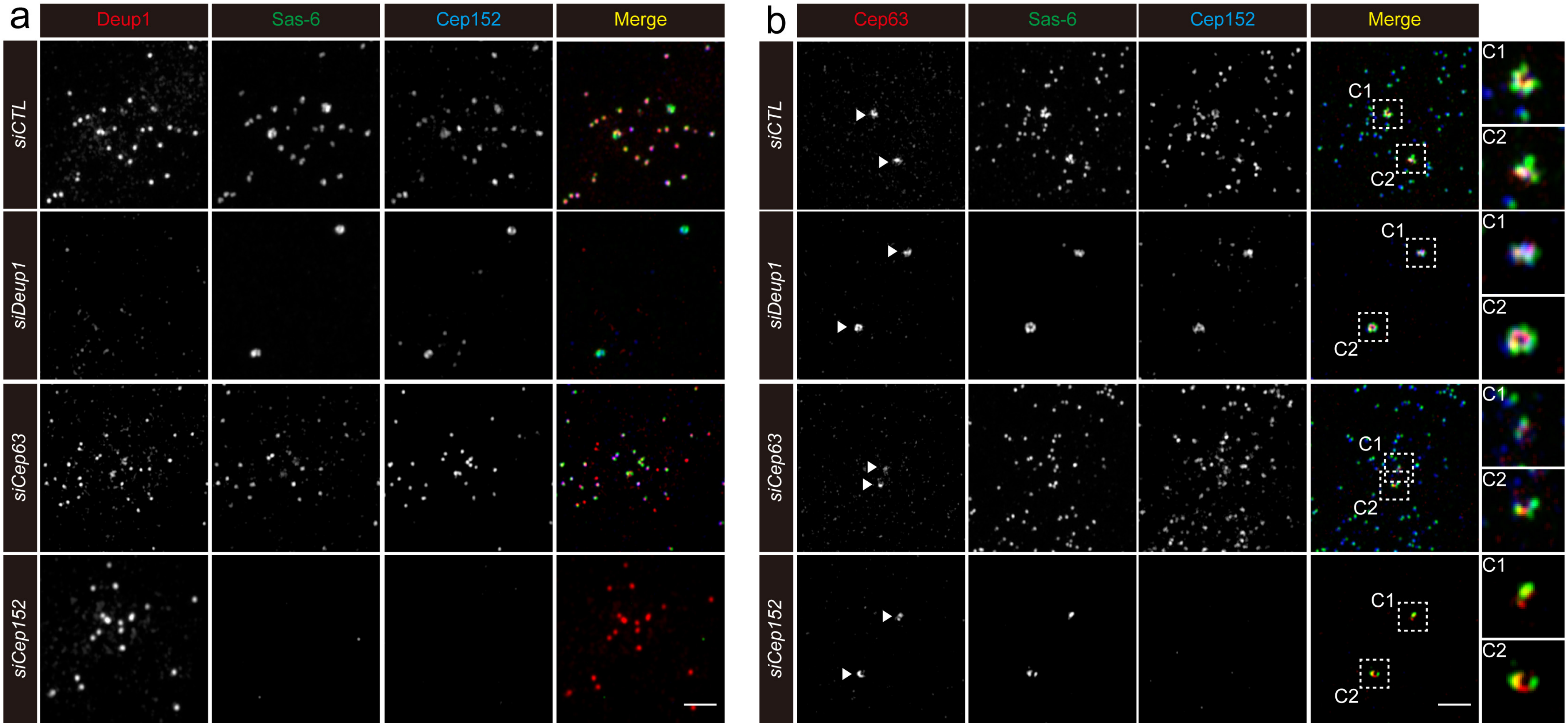

Kim et al Figure S7

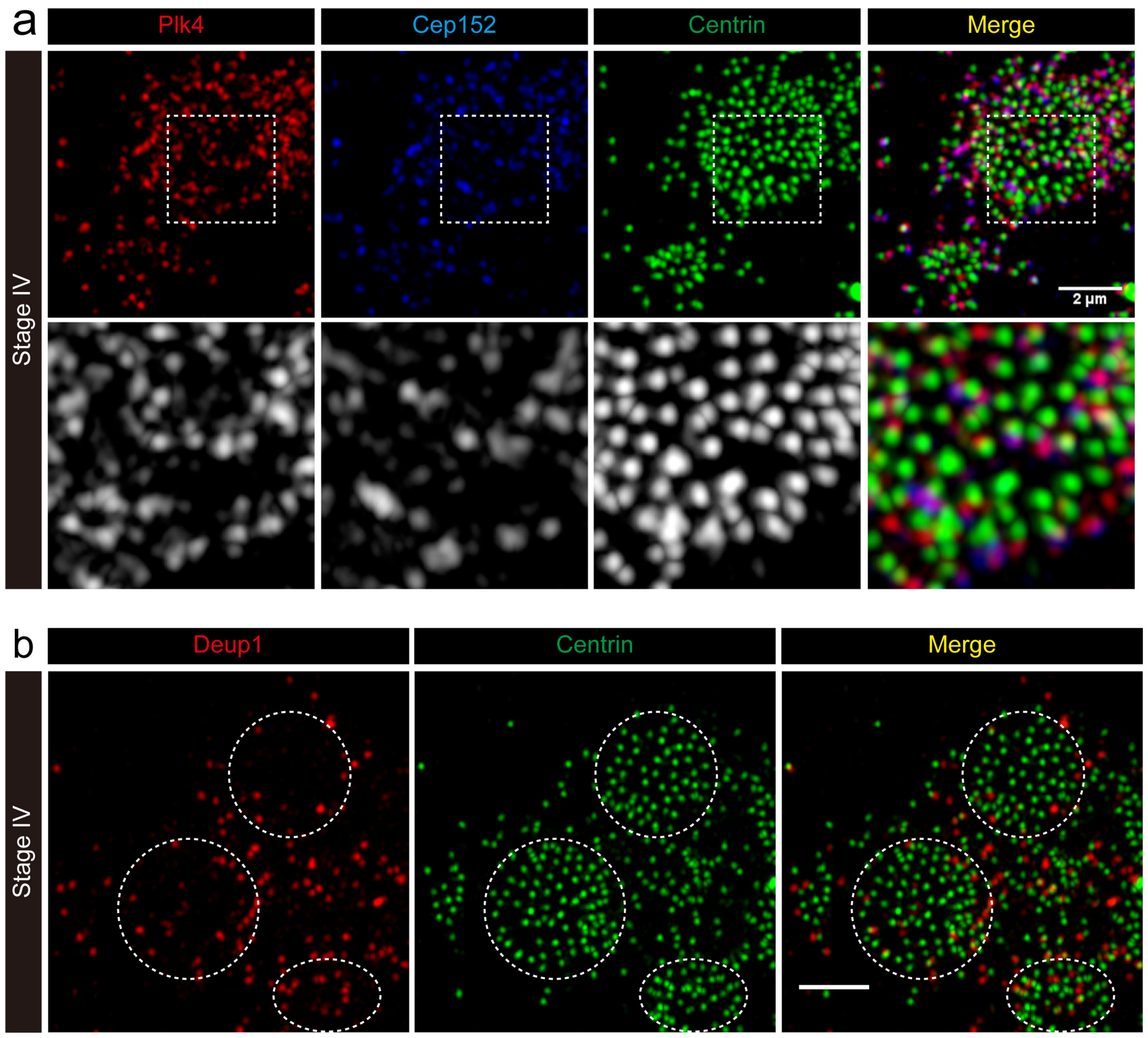

Kim et al Figure S8

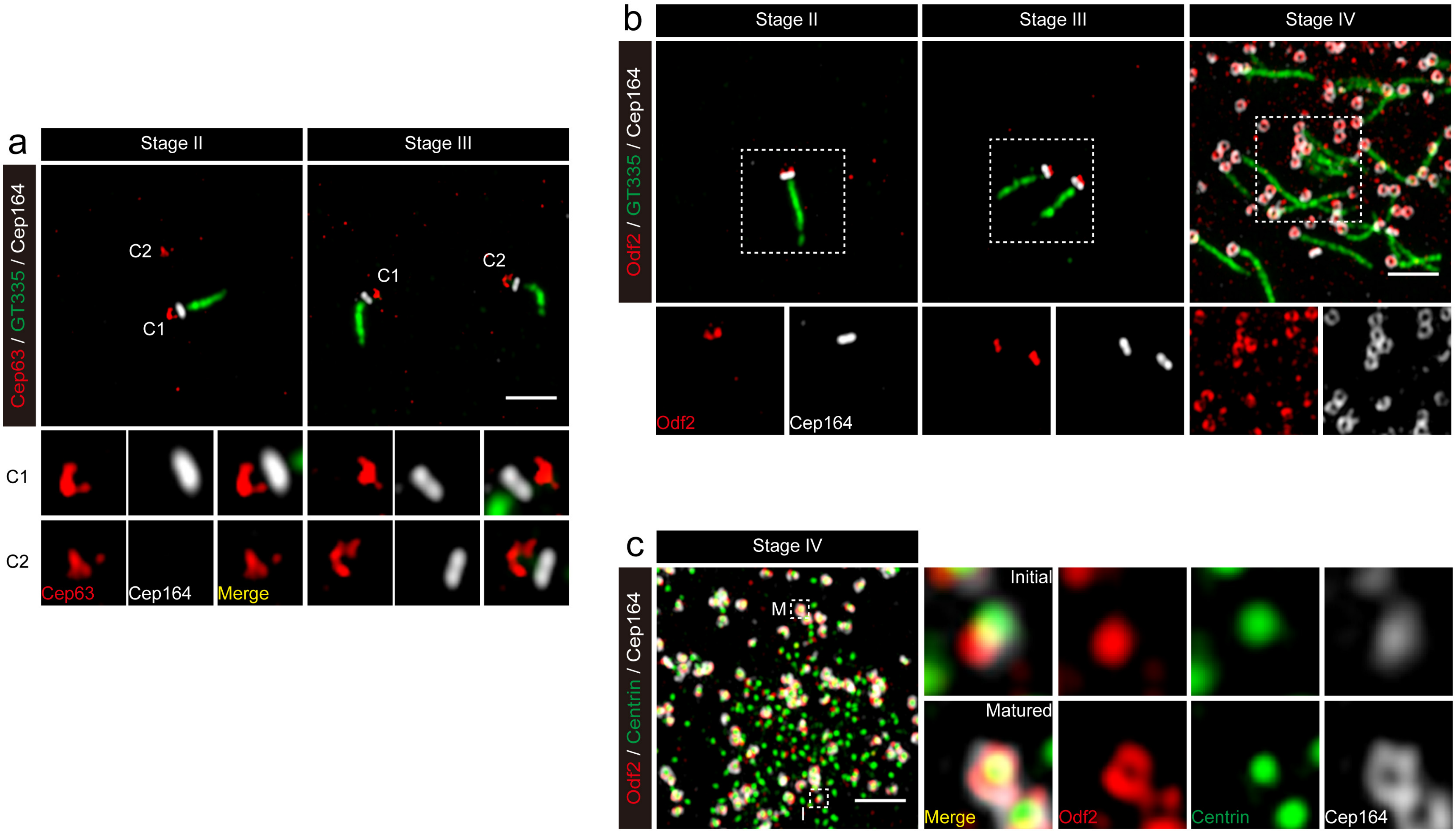

Kim et al Figure S9

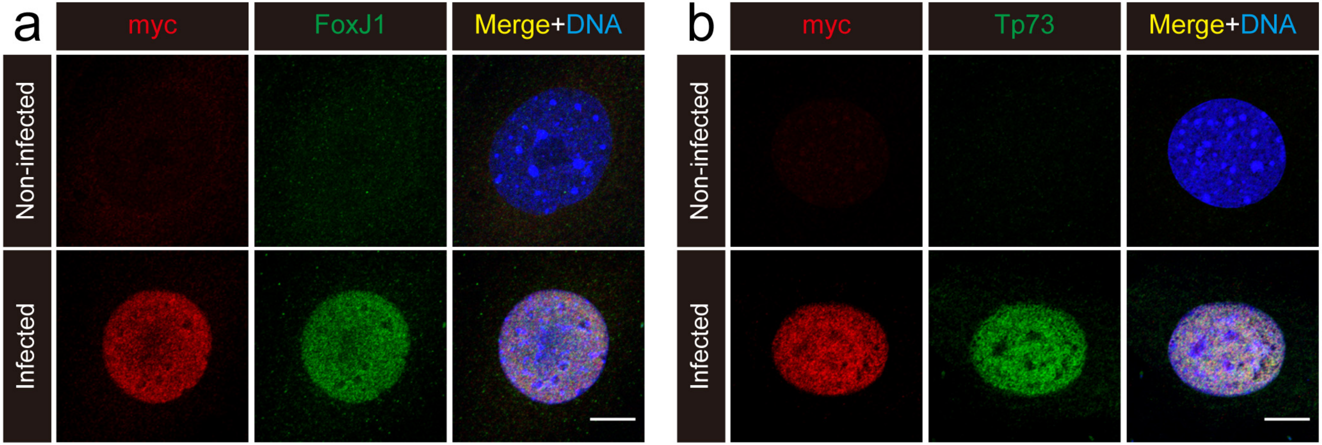

c

	Foxj1 expression	Tp73 expression
Non-infected cell (%)	0%	0%
Infected cell (%) (Foxj1+ or Tp73+ / myc+ cell)	81% (204/252)	88% (215/245)

Kim et al Figure S10

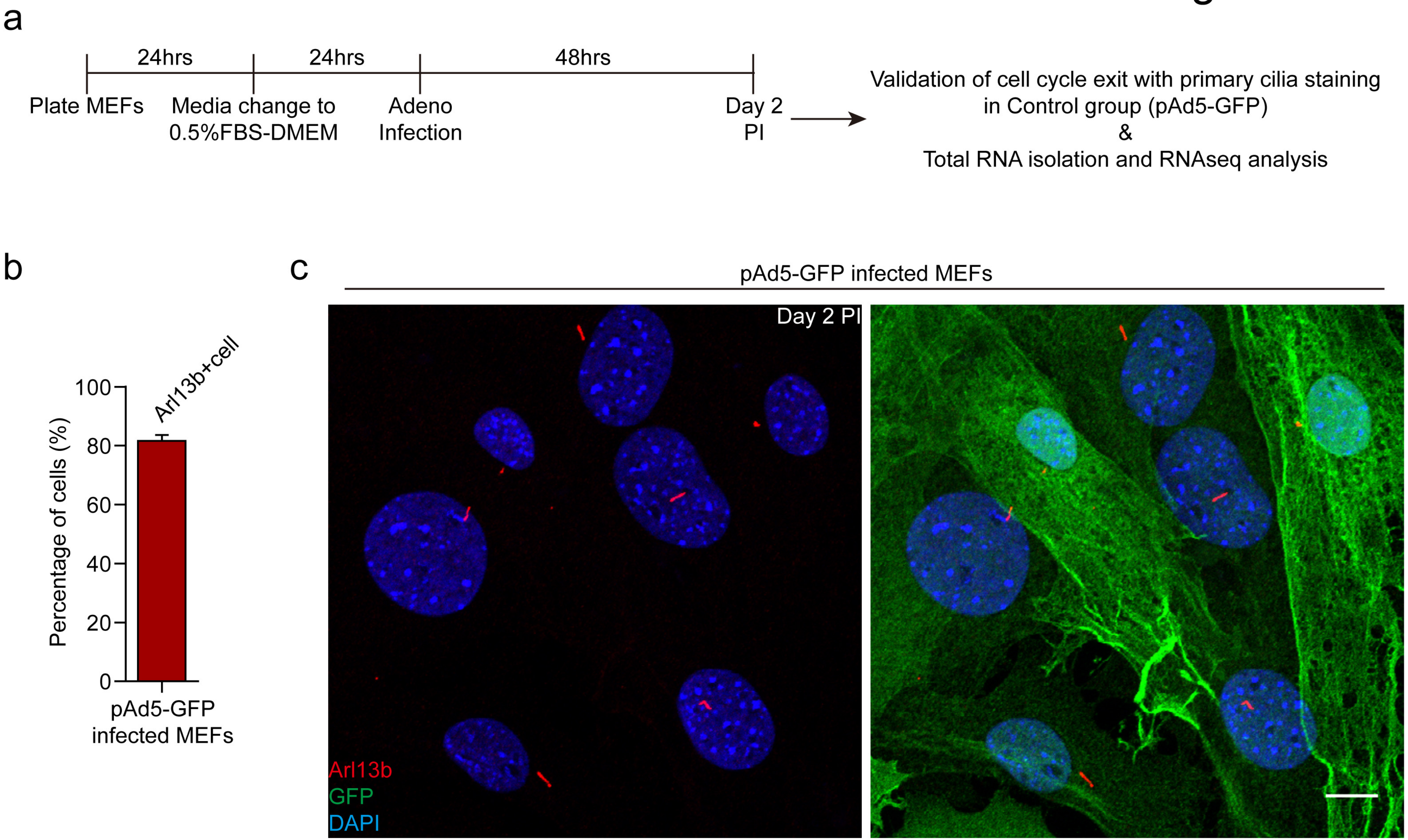

Kim et al Figure S11

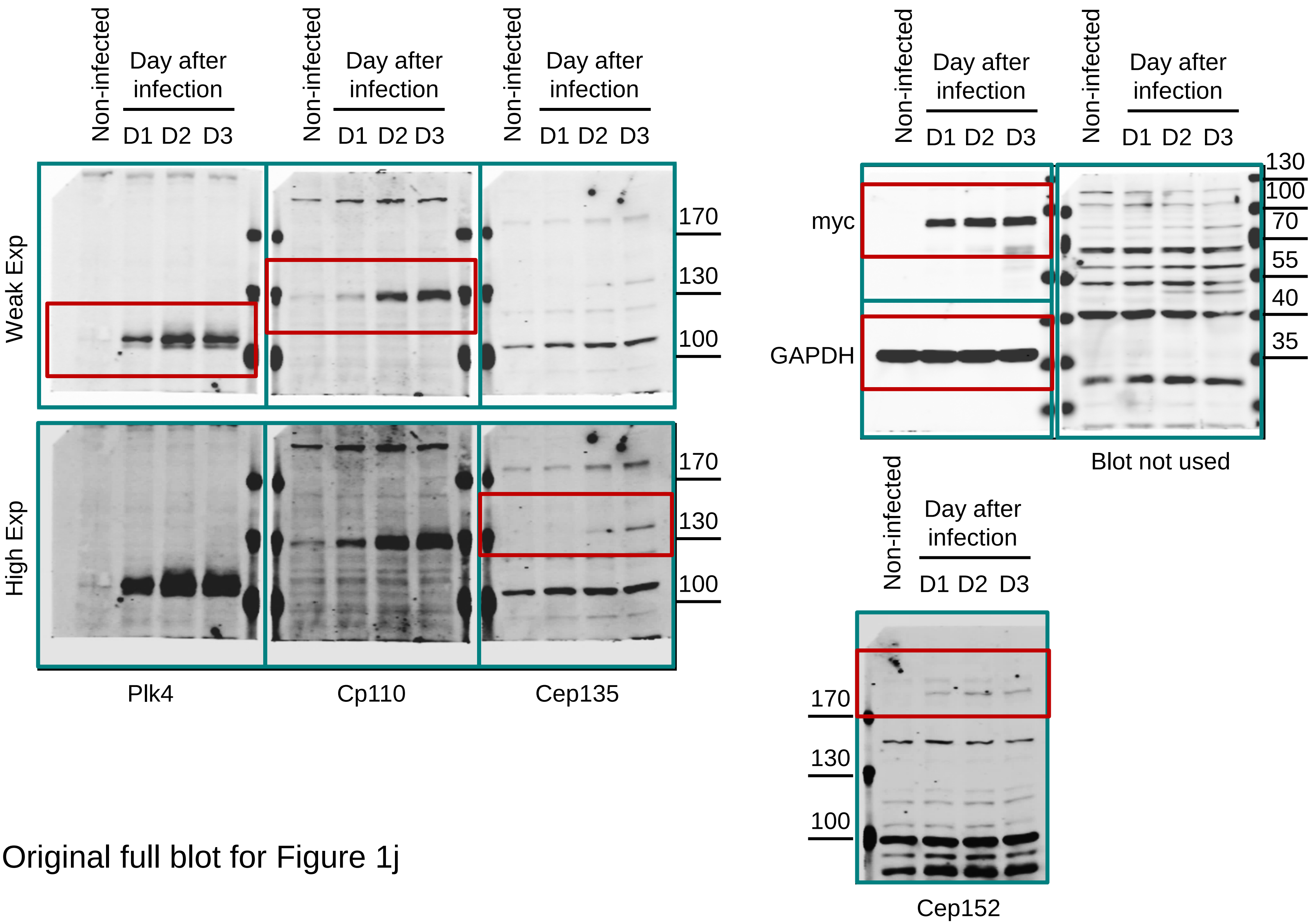

Original full blot for Figure 1j

Kim et al Figure S12

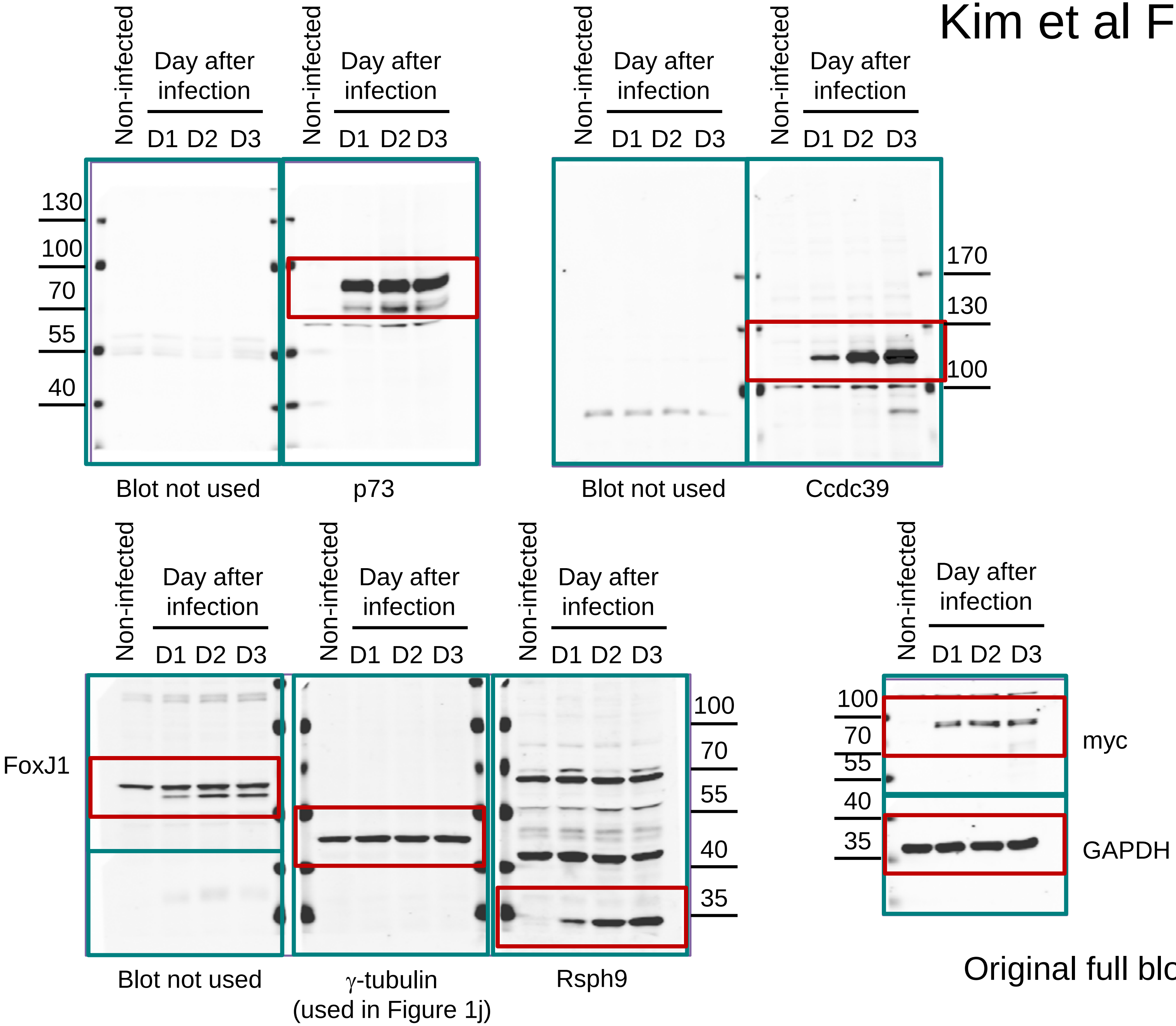

Original full blot for Figure 4b

Kim et al Figure S13

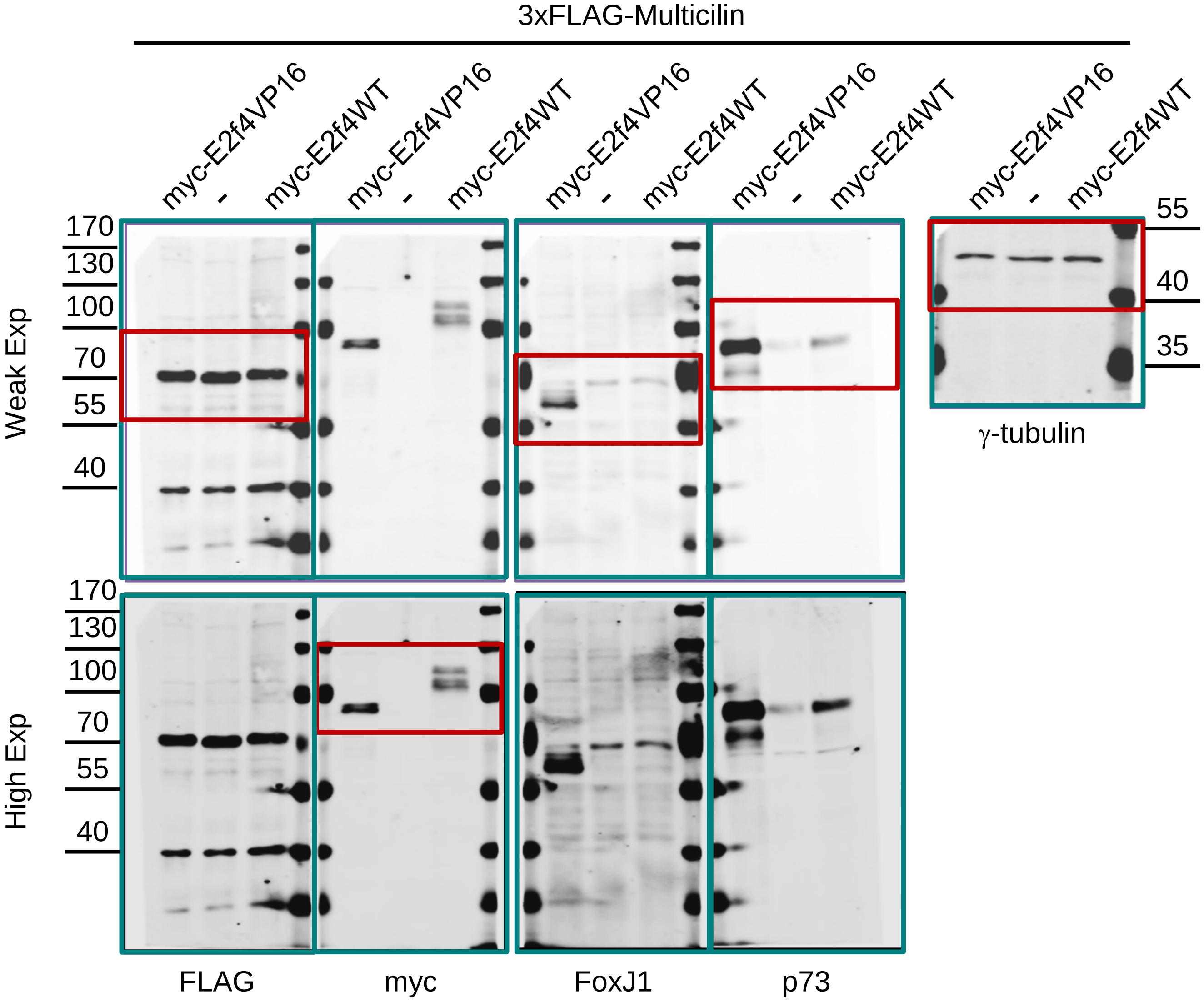

Original full blot for Figure S2c

Kim et al Figure S14

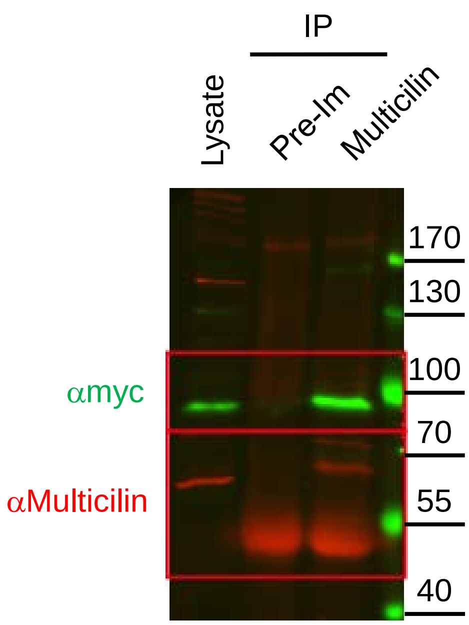

mouse α myc / anti-mouse Alexa680 (green)
rabbit α Multicilin / anti-rabbit Alexa800 (red)

Original full blot for Fig S3b

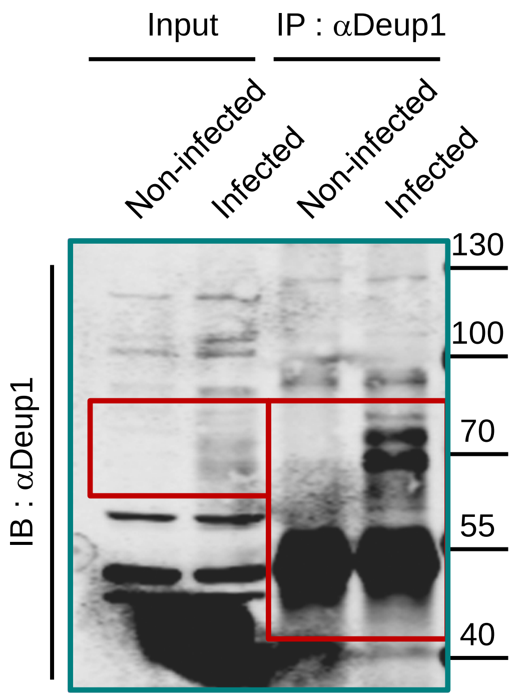

Original full blot for Fig S4c
